# MATRix–RICE therapy and autologous haematopoietic stem-cell transplantation in diffuse large B-cell lymphoma with secondary CNS involvement (MARIETTA): an international, single-arm, phase 2 trial

**DOI:** 10.1016/S2352-3026(20)30366-5

**Published:** 2021-01-26

**Authors:** Andrés J M Ferreri, Jeanette K Doorduijn, Alessandro Re, Maria Giuseppina Cabras, Jeffery Smith, Fiorella Ilariucci, Mario Luppi, Teresa Calimeri, Chiara Cattaneo, Jahanzaib Khwaja, Barbara Botto, Claudia Cellini, Luca Nassi, Kim Linton, Pam McKay, Jacopo Olivieri, Caterina Patti, Francesca Re, Alessandro Fanni, Vikram Singh, Jacoline E C Bromberg, Kelly Cozens, Elisabetta Gastaldi, Massimo Bernardi, Nicola Cascavilla, Andrew Davies, Christopher P Fox, Maurizio Frezzato, Wendy Osborne, Anna Marina Liberati, Urban Novak, Renato Zambello, Emanuele Zucca, Kate Cwynarski

**Affiliations:** a Lymphoma Unit, Department of Onco-Hematology, IRCCS San Raffaele Scientific Institute, Milan, Italy; b Department of Hematology, Erasmus MC Cancer Institute, Rotterdam, Netherlands; c Department of Neurology, Erasmus MC Cancer Institute, Rotterdam, Netherlands; d Spedali Civili, Brescia, Italy; e Ospedale Oncologico Businco, Cagliari, Italy; f Department of Haematology, Aintree Hospital, Liverpool, UK; g Division of Hematology, Azienda USL-IRCCS Reggio Emilia, Reggio Emilia, Italy; h Azienda Ospedaliera Universitaria, UNIMORE, Modena, Italy; i Department of Haematology, University College Hospital, London, UK; j AOU Città della salute e della Scienza, Turin, Italy; k Ospedale di Ravenna, IRST, Ravenna, Italy; l Division of Hematology, Department of Translational Medicine, University of Eastern Piedmont and AOU Maggiore della Carità, Novara, Italy; m Department of Haemato-Oncology, The Christie Hospital, Manchester, UK; n Beatson Cancer Centre, Glasgow, UK; o AOU Santa Maria della Misericordia, Udine, Italy; p Division of Hematology, Azienda Villa Sofia-Cervello, Palermo, Italy; q AO Parma, Parma, Italy; r Southampton Clinical Trials Unit, University of Southampton, Southampton, UK; s Cancer Research UK Centre, University of Southampton, Southampton, UK; t International Extranodal Lymphoma Study Group Coordinating Center, Institute of Oncology Research, Università della Svizzera Italiana, Bellinzona, Switzerland; u Casa Sollievo della Sofferenza Hospital, IRCCS, San Giovanni Rotondo, Italy; v Department of Clinical Haematology, Nottingham University Hospitals NHS Trust, Nottingham, UK; w San Bortolo Hospital, Vicenza, Italy; x Department of Haematology, Newcastle Upon Tyne Hospitals NHS Foundation Trust, Newcastle, UK; y Università Degli Studi di Perugia—AO Santa Maria, Terni, Italy; z Department of Medical Oncology, Inselspital, Bern University Hospital, Bern, Switzerland; aa Azienda Ospedaliera di Padova, Padua, Italy; ab Medical Oncology Clinic, Oncology Institute of Southern Switzerland, Ente Ospedaliero Cantonale, Bellinzona, Switzerland; ac Swiss Group for Clinical Cancer Research, Bern, Switzerland; ad Faculty of Biomedical Sciences, Università della Svizzera Italiana, Lugano, Switzerland

## Abstract

**Background:**

Secondary CNS lymphoma is a rare but potentially lethal event in patients with diffuse large B-cell lymphoma. We aimed to assess the activity and safety of an intensive, CNS-directed chemoimmunotherapy consolidated by autologous haematopoietic stem-cell transplantation (HSCT) in patients with secondary CNS lymphoma.

**Methods:**

This international, single-arm, phase 2 trial was done in 24 hospitals in Italy, the UK, the Netherlands, and Switzerland. Adults (aged 18–70 years) with histologically diagnosed diffuse large B-cell lymphoma and CNS involvement at the time of primary diagnosis or at relapse and Eastern Cooperative Oncology Group Performance Status of 3 or less were enrolled and received three courses of MATRix (rituximab 375 mg/m^2^, intravenous infusion, day 0; methotrexate 3·5 g/m^2^, the first 0·5 g/m^2^ in 15 min followed by 3 g/m^2^ in a 3 h intravenous infusion, day 1; cytarabine 2 g/m^2^ every 12 h, in 1 h intravenous infusions, days 2 and 3; thiotepa 30 mg/m^2^, 30 min intravenous infusion, day 4) followed by three courses of RICE (rituximab 375 mg/m^2^, day 1; etoposide 100 mg/m^2^ per day in 500–1000 mL over a 60 min intravenous infusion, days 1, 2, and 3; ifosfamide 5 g/m^2^ in 1000 mL in a 24 h intravenous infusion with mesna support, day 2; carboplatin area under the curve of 5 in 500 mL in a 1 h intravenous infusion, day 2) and carmustine–thiotepa and autologous HSCT (carmustine 400 mg/m^2^ in 500 mL glucose 5% solution in a 1–2 h infusion, day −6; thiotepa 5 mg/kg in saline solution in a 2 h infusion every 12 h, days −5 and −4). The primary endpoint was progression-free survival at 1 year. Overall and complete response rates before autologous HSCT, duration of response, overall survival, and safety were the secondary endpoints. Analyses were in the modified intention-to-treat population. This study is registered with ClinicalTrials.gov, NCT02329080. The trial ended after accrual completion; the database lock was Dec 31, 2019.

**Findings:**

Between March 30, 2015, and Aug 3, 2018, 79 patients were enrolled. 75 patients were assessable. 319 (71%) of the 450 planned courses were delivered. At 1 year from enrolment the primary endpoint was met, 42 patients were progression free (progression-free survival 58%; 95% CI 55–61). 49 patients (65%; 95% CI 54–76) had an objective response after MATRix–RICE, 29 (39%) of whom had a complete response. 37 patients who responded had autologous HSCT. At the end of the programme, 46 patients (61%; 95% CI 51–71) had an objective response, with a median duration of objective response of 26 months (IQR 16–37). At a median follow-up of 29 months (IQR 20–40), 35 patients were progression-free and 33 were alive, with a 2-year overall survival of 46% (95% CI 39–53). Grade 3–4 toxicity was most commonly haematological: neutropenia in 46 (61%) of 75 patients, thrombocytopenia in 45 (60%), and anaemia in 26 (35%). 79 serious adverse events were recorded in 42 (56%) patients; four (5%) of those 79 were lethal due to sepsis caused by Gram-negative bacteria (treatment-related mortality 5%; 95% CI 0·07–9·93).

**Interpretation:**

MATRix–RICE plus autologous HSCT was active in this population of patients with very poor prognosis, and had an acceptable toxicity profile.

**Funding:**

Stand Up To Cancer Campaign for Cancer Research UK, the Swiss Cancer Research foundation, and the Swiss Cancer League.

## Introduction

Secondary CNS dissemination is a rare but potentially lethal event in patients with diffuse large B-cell lymphoma. CNS dissemination might present de novo, synchronously with systemic disease, or as early CNS relapse (isolated or with concomitant systemic disease).^1^, ^2^, ^3^, ^4^ Secondary CNS lymphoma involves the brain parenchyma in 40–50% of patients, leptomeninges in 30–40%, and both in 10–15%, but the prevalence and timing of these events vary due to the diversity of CNS prophylaxis strategies.^1^, ^5^ Neurological symptoms are the first indication of CNS disease in many patients, and clinical presentation is influenced by the site of the CNS lesions.^6^ Secondary CNS lymphoma is frequently associated with systemic lymphoma progression, concomitantly or shortly afterwards; thus, its treatment should be effective against both the systemic and the CNS components of the disease. Rationally designed treatment protocols should use drugs that can be delivered in a dose-intensive and time-intensive schedule and are effective at penetrating the blood–brain barrier, such as methotrexate and cytarabine administered at high doses. On the basis of retrospective studies,^7^, ^8^ it is believed that potential cure is only achievable if patients have autologous haematopoietic stem-cell transplantation (HSCT) consolidation after remission of CNS disease. Accordingly, conditioning regimens currently used in primary CNS lymphoma, which include drugs with excellent CNS penetration, such as carmustine, busulfan, and thiotepa,^9^ have been adopted in patients with secondary CNS lymphoma.

**Research in context**

**Evidence before this study**

We searched PubMed between Jan 1, 1990, and Dec 31, 2019, for prospective trials investigating the treatment of patients with secondary CNS lymphoma. We used the search terms “central nervous system”, “CNS”, “lymphoma”, “secondary CNS lymphoma”, “SCNSL”, AND “diffuse large B-cell lymphoma”, and restricted the results to articles published in English. Publications in non-English languages with abstracts in English were also considered. Abstract-only data from international meetings during the past 3 years (up to Dec 31, 2019) were considered. There is little evidence in this field; secondary CNS lymphoma is a rare lymphoma and randomised trials are not feasible, hence the paucity of data to inform practice. Existing literature comprises only three single-arm phase 2 trials including at most 38 patients. These are non-comparable trials as they have used different eligibility criteria with variation in the upper limits of age and performance status as well as in considered lymphoma entities, previous treatment, and response status in the selection of candidates for autologous haematopoietic stem-cell transplantation (HSCT). In addition, data on some subgroups, such as patients with CNS involvement at initial diagnosis and patients with disease refractory to the first-line treatment, are scarce because these patients were considered in only one of the published prospective studies and accounted for less than a fifth of the patients enrolled. The reviewed literature does not allow the identification of a standard treatment for patients with secondary CNS lymphoma.

**Added value of this study**

To our knowledge, the MARIETTA study is the largest prospective trial focused on patients with secondary CNS lymphoma; it was done in 24 centres in four countries, representing the most geographically extensive trial to date, which supports the generalisability of the results. This trial showed that the sequential combination of MATRix (rituximab, methotrexate, cytarabine, and thiotepa) and RICE (rituximab, ifosfamide, carboplatin, and etoposide) followed by autologous HSCT was active in this population of patients with a very poor prognosis. Patients aged up to 70 years and with an Eastern Cooperative Oncology Group Performance Status of 3 or less were included, representing a real-world cohort of patients. The MARIETTA programme is active in every subgroup of secondary CNS lymphoma, with 1-year progression-free survival of 58% (95% CI 55–61) and encouraging 2-year progression-free survival of 71% (63–79) in patients with CNS disease at initial lymphoma diagnosis. Moreover, the MARIETTA programme included two standardised regimens (ie, MATRIX and RICE) used in routine practice in several countries, and showed a good safety profile.

**Implications of all the available evidence**

Combined with existing evidence, the results of the MARIETTA trial show that a growing proportion of patients with secondary CNS lymphoma can have durable remissions with intensified chemoimmunotherapy. Patients with CNS disease at initial lymphoma diagnosis and patients who had CNS dissemination during or after upfront R-CHOP therapy have different outcomes, suggesting that these two populations of patients with secondary CNS lymphoma might benefit from different treatments. Patients with CNS involvement at initial diagnosis seemed to benefit from treatment with debulking R-CHOP followed by MATRix–RICE and autologous HSCT. Further efforts are required to improve remission rates before autologous HSCT, especially in patients with CNS involvement at first relapse.

The highest level of evidence in the treatment of secondary CNS lymphoma is from three single-arm phase 2 trials on 30–38 patients each.^10^, ^11^, ^12^ These trials are non-comparable because they used different eligibility criteria with variation in upper limits of age, performance status, included lymphoma subtypes, previous treatment, and response status in the selection of patients for autologous HSCT. Additionally, data on some secondary CNS lymphoma subgroups, such as patients with CNS involvement at presentation and patients with disease refractory to the first-line treatment, are scarce because these patients were considered in only one of the published prospective studies and accounted for less than a fifth of the patients enrolled.^11^

We therefore designed the MARIETTA trial, which aimed to assess an intensive, CNS-directed chemoimmunotherapy consolidated by autologous HSCT in patients with secondary CNS lymphoma. Here, we report safety and activity results of this trial.

## Methods

### Study design and participants

The MARIETTA trial is an international, single-arm, phase 2 trial done in 24 hospitals in four countries (Italy, the UK, the Netherlands, and Switzerland; appendix p 2). The trial assessed the safety and activity of a sequential combination of MATRix (methotrexate, cytarabine, thiotepa, and rituximab)^13^ followed by RICE (rituximab, ifosfamide, carboplatin, and etoposide) and carmustine–thiotepa conditioned autologous HSCT in patients with diffuse large B-cell lymphoma and secondary CNS involvement at diagnosis or relapse. These treatment combinations were selected following the positive results in primary CNS lymphoma^14^, ^15^, ^16^ and relapsed or refractory diffuse large B-cell lymphoma^17^ (rationale shown in appendix p 4).

The main inclusion criteria were: histologically proven diagnosis of diffuse large B-cell lymphoma; CNS involvement (brain, meninges, cranial nerves, eyes, spinal cord, or a combination of these) at presentation (concomitant to systemic disease) or at relapse (isolated or concomitant to systemic lymphoma); age 18–70 years; Eastern Cooperative Oncology Group Performance Status (ECOG-PS) of 3 or less; and no previous treatment with high-dose methotrexate. Both previously untreated patients with CNS involvement at initial diagnosis and patients with CNS relapse during or after a treatment for systemic lymphoma were included. Any previous treatment for systemic lymphoma except for high-dose methotrexate or autologous or allogeneic HSCT was admitted. The upper age limit reflected the eligibility of patients to receive autologous HSCT in the participating centres. Diagnosis of CNS involvement by either brain biopsy, cerebrospinal fluid cytology examination, or neuroimaging was permitted. Patients with high-grade transformation from indolent lymphoma and patients with double-hit or triple-hit lymphoma were eligible. Patients with a previous organ transplantation or other forms of immunosuppression, HIV infection, or primary CNS lymphoma were excluded. Staging and pretreatment tests (appendix p 5) were done within 14 days of the start of treatment. A full list of inclusion and exclusion criteria is in the appendix (p 3). Sociocultural information and data on ethnicity were not collected. Written informed consent was obtained from each patient. This trial conformed to the Declaration of Helsinki and was approved by the institutional review boards of the participating institutions. The protocol is included in the appendix (p 23).

### Procedures

After enrolment (appendix p 13), patients received three courses of MATRix^13^ followed by three courses of RICE, each delivered every 3 weeks. Patients enrolled at initial lymphoma diagnosis (chemotherapy-naive patients) with extensive and life-threatening extra-CNS disease received one or two courses of R-CHOP (rituximab 375 mg/m^2^ as an intravenous infusion on day 1; cyclophosphamide 750 mg/m^2^ as an intravenous bolus on day 1; doxorubicin 50 mg/m^2^ as an intravenous bolus on day 1; vincristine 1·4 mg/m^2^ [maximum 2 mg] as an intravenous bolus on day 1; prednisone 75 mg per day orally on days 1–5). The MATRix regimen was rituximab 375 mg/m^2^ as an intravenous infusion on day 0; methotrexate 3·5 g/m^2^, the first 0·5 g/m^2^ in 15 min followed by 3 g/m^2^ as a 3 h intravenous infusion on day 1; cytarabine 2 g/m^2^ every 12 h, in 1 h intravenous infusions on days 2 and 3; and thiotepa 30 mg/m^2^ in a 30 min intravenous infusion on day 4.^13^ The RICE regimen was rituximab 375 mg/m^2^ on day 1; etoposide 100 mg/m^2^ per day in 500–1000 mL over a 1 h intravenous infusion on days 1, 2, and 3; ifosfamide 5 g/m^2^ in 1000 mL in a 24 h intravenous infusion with mesna support on day 2; and carboplatin area under the curve of 5 in 500 mL in a 1 h intravenous infusion on day 2. Intrathecal chemotherapy was indicated in every enrolled patient regardless of cerebrospinal fluid cytology status because conventional cytology examination is associated with frequent false negative results, particularly when flow cytometry is not routinely used.^6^ Accordingly, a dose of intrathecal liposomal cytarabine 50 mg or conventional triple-drug chemotherapy (intrathecal methotrexate 12 mg, cytarabine 50 mg, and hydrocortisone 50 mg) was delivered on day 5 of every course of MATRix and on day 4 of every course of RICE. Per protocol, antimicrobial prophylaxis followed institutional guidelines; however, oral antiviral, antifungal, and antipneumocystic prophylaxis plus conventional doses of recombinant granulocyte-colony stimulating factor (G-CSF; exact molecule chosen by investigators) from day 6 to 12 of every chemotherapy course was suggested.

The protocol mandated that patients with stable or progressive disease during MATRix treatment would immediately switch to the RICE regimen (appendix p 13), with patients with subsequent CNS progression events receiving whole-brain radiotherapy before proceeding with autologous HSCT consolidation. The whole-brain radiotherapy dose was 36 Gy plus a tumour-bed boost of 10 Gy. The whole brain was irradiated by two opposite lateral fields, including the first two cervical vertebrae and the posterior two-thirds of the eyes, which had to be shielded after 30 Gy (photons of 4–10 MeV, 180–200 cGy per day, five weekly fractions). Autologous peripheral blood stem cells were collected after the second course of MATRix and were processed according to conventional guidelines. Patients with a complete or partial response after MATRix–RICE and with adequate autologous peripheral blood stem cell harvest received autologous HSCT (appendix p 13). Myeloablative chemotherapy consisted of carmustine 400 mg/m^2^ in 500 mL glucose 5% solution in a 1–2 h infusion on day −6; thiotepa 5 mg/kg in saline solution in 2 h infusions every 12 h on day −5 and −4, supported by autologous HSCT. In case of carmustine unavailability, the recommended conditioning regimen was: thiotepa 5 mg/kg in 100 mL of saline solution by 2 h infusion on day −6 and −5; busulfan 3·2 mg/kg (administered in four doses per day corresponding to 0·8 mg/kg each dose) by 2 h infusion or 3·2 mg/kg as a once daily infusion given over 3 h, on days −4, −3, and −2. Patients with residual disease in the brain parenchyma after autologous HSCT received whole-brain radiotherapy; patients with residual disease in the cerebrospinal fluid after autologous HSCT received additional, intensified intrathecal chemotherapy (methotrexate 12 mg plus cytarabine 50 mg plus hydrocortisone 50 mg on days 1 and 8 each month for 3 months, or thiotepa 10 mg plus rituximab 25 mg on days 4 and 11 each month for 3 months, or both).

Toxicities were graded according to the National Cancer Institute–National Cancer Institute of Canada Common Toxicity Criteria (NCI–NCIC CTC) version 4.0.^18^ The worst toxicity per organ, per course was considered. Adverse events were assessed during the visit before each course or during hospitalisation, when required. Severe adverse events are defined in the protocol (appendix p 23). Tumour response was assessed by gadolinium-enhanced brain MRI, ^18^F-fluorodeoxyglucose PET, contrast-enhanced total-body CT scan, and other examinations that were positive at baseline, after the second course of MATRix, the first course of RICE, the third course of RICE, and after autologous HSCT. Response definition of CNS disease followed the International Primary CNS Lymphoma Collaborative Group response criteria^19^ and extra-CNS disease followed the Revised Response Criteria for Malignant Lymphoma.^20^ The maximum response recorded from treatment start was considered for analyses and therapeutic decisions were based on local investigator assessment. After the end of treatment, the disease was assessed every 3 months for the first 2 years, and every 6 months during the third, fourth, and fifth years.

### Outcomes

The primary endpoint was progression-free survival at 1 year. The secondary endpoints were overall and complete response rate before autologous HSCT, duration of response, overall survival, and safety. Progression-free survival and overall survival were estimated according to the Revised Response Criteria for Malignant Lymphoma.^20^ The best response achieved during experimental treatment was considered for analyses, and duration of response was estimated for patients who had a response to MATRix–RICE as the time between the date of best response and the date of relapse, progressive disease, death from any cause, or last follow-up visit. Stable disease during MATRix that required crossing to RICE, or stable disease during RICE that required crossing to whole-brain radiotherapy, was not considered an event in survival analyses. The effects of treatment on patient-reported outcomes (acceptability and quality of life) were not assessed.

Post-hoc exploratory analyses were to define differences in progression-free survival between patients with CNS localisation at initial diagnosis and patients with CNS involvement at relapse, difference in progression-free survival between patients with histologically confirmed CNS disease and patients assessed with neuroimaging alone, the role of debulking R-CHOP and intrathecal chemotherapy, and the prognostic value of age, sex, performance status, lactate dehydrogenase serum concentration, sites of disease, response to the first two MATRix courses, a history of indolent lymphoma, and cell of origin.

### Statistical analysis

The Fleming design was used. The maximum progression-free survival at 1 year considered of low interest was 50% (null hypothesis)^10^ and the minimum progression-free survival at 1 year considered of interest was 65% (alternative hypothesis). In other words, an experimental treatment associated with a 1-year progression-free survival of less than 50% would not be a suitable strategy for routine use and further research. To detect such a difference 69 patients were required (one-sided test, type I error 5%, and power 80%); with a 10% dropout, 76 patients were needed. If at least 41 patients were progression-free survivors at 1 year, the strategy would be considered effective. All registered patients were considered for primary and safety analyses except for patients who post hoc objectively did not meet the eligibility criteria, including incorrect histopathological diagnosis, concomitant cancer, or disease only at flow cytometry examination of the cerebrospinal fluid (modified intention-to-treat analysis). Patients were excluded from analyses in the case of consensus withdrawal.

Progression-free survival and overall survival curves were generated using the Kaplan-Meier method, expressed with standard error, and compared through the log-rank test. 95% CI was provided for any data derived from Kaplan-Meier analyses. Time at risk started at the day of trial enrolment for all the analyses except the analysis of response to MATRix, for which the time at risk started at the date of response assessment after the first two MATRix courses.

Independent association between studied variables and survival were tested using the Cox proportional hazard model. Variables that achieved statistical significance (p<0·05) in univariate analyses were considered for the multivariable analysis. Variables addressed by unplanned exploratory analyses were not included in the multivariable analysis. All the probability values were two-sided. All analyses were done using the Statistica 10.0 statistical package for Windows (Statsoft, 2011, Tulsa, OK, USA). This study was registered with ClinicalTrials.gov, NCT02329080.

### Role of the funding source

Neither the sponsor nor the grant provider for the study had any role in study design, data collection, data analysis, data interpretation, or writing of the report. All the authors had full access to all the data in the study and had final responsibility for the decision to submit for publication.

## Results

**Figure 1:**
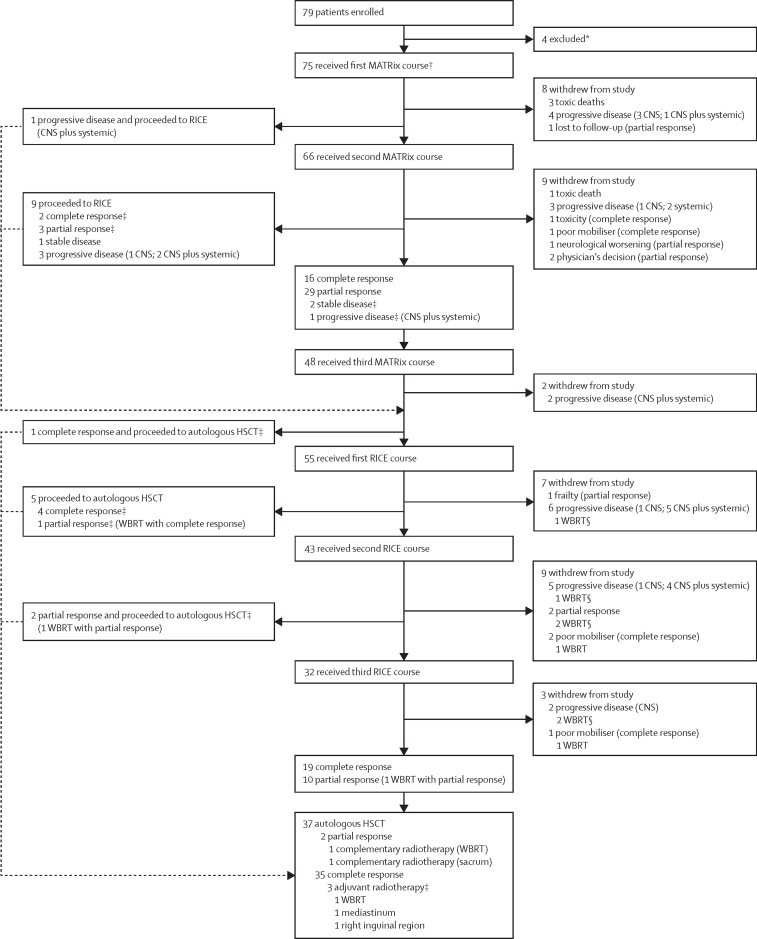
Trial profile HSCT=high-dose chemotherapy supported by autologous haematopoietic stem-cell transplantation. MATRix=rituximab, methotrexate, cytarabine, and thiotepa. RICE=rituximab, ifosfamide, carboplatin, and etoposide. WBRT=whole-brain radiotherapy. Adjuvant radiotherapy is radiotherapy used in patients in complete remission after autologous HSCT. Complementary radiotherapy is irradiation of residual lesions in patients in partial response after autologous HSCT. *Four patients were excluded because of unrelated laboratory abnormalities (n=2), disease only at flow cytometry examination of the cerebrospinal fluid (n=1), and death at the same time as registration (n=1). †Per protocol, MATRix was preceded by debulking R-CHOP (rituximab, cyclophosphamide, doxorubicin, vincristine, and prednisone) in nine (28%) of the 32 patients enrolled at original lymphoma diagnosis. ‡Protocol deviations. §Patients still had progressive disease after the WBRT.

Between March 30, 2015, and Aug 3, 2018, 79 patients were enrolled. Every patient who was eligible according to the per-protocol selection criteria and diagnosed at participating centres was enrolled. The trial was ended after accrual completion; the database lock was Dec 31, 2019. Four patients were excluded after enrolment before the start of study treatment because of unrelated laboratory abnormalities (two patients), disease only at flow cytometry examination of the cerebrospinal fluid (one), and death at the same time as registration (one). 75 patients with a median age of 58 years (IQR 50–66) were assessable and received the first MATRix course (figure 1), 38 (51%) of whom were male and 37 (49%) of whom were female (table 1; appendix p 6).

**Table 1:** Baseline characteristics

		**Patients (n=79)**
Eligible patients (study population)		75
Age, years		58 (50–66; 23–70)
Sex		
	Male	38 (51%)
	Female	37 (49%)
Disease at enrolment		
	CNS involvement at presentation	32 (43%)
	Isolated CNS relapse	15 (20%)
	Concomitant CNS–systemic localisation	28 (37%)
HBV or HCV seropositivity		2 (3%)
Symptoms at enrolment		
	B symptoms*	6 (8%)
	Motor impairment	37 (49%)
	Sensorial impairment	25 (33%)
	Language impairment	7 (9%)
	Cognitive impairment	15 (20%)
	Sensorial impairment	12 (16%)
CNS sites of disease		
	Brain parenchyma	34 (45%)
	Cerebrospinal fluid or meninges	8 (11%)
	Spinal cord	2 (3%)
	Eyes	2 (3%)
	Brain and cerebrospinal fluid or meninges	13 (17%)
	Brain and eyes	10 (13%)
	Brain, cerebrospinal fluid, and eyes	6 (8%)
Extra-CNS sites of disease at enrolment		
	None	15 (20%)
	Nodal disease (only lymphadenopathies)	18 (24%)
	Extranodal disease (other than CNS)	18 (24%)
	Nodal and extranodal disease	24 (32%)
Extranodal disease at initial lymphoma diagnosis†		59 (79%)
IPI		
	Age >60 years	32 (43%)
	ECOG-PS >1‡	28 (37%)
	Number of extranodal organs involved >1 (other than CNS)	23 (31%)
	High LDH serum concentration	37 (49%)
	Advanced stage	60 (80%)
	Low IPI risk	14 (19%)
	Low–intermediate IPI risk	18 (24%)
	High–intermediate IPI risk	26 (35%)
	High IPI risk	17 (23%)
Previous treatment		43 (57%)
	R-CHOP	40 (93%)
	Other doxorubicin–rituximab (DA-R-EPOCH, R-VACOP)	2 (5%)
	Rituximab–bendamustine	1 (2%)
	Previous CNS prophylaxis (only intrathecal chemotherapy)	7 (16%)
	Refractory to previous treatment (n=43)	20 (47%)

Data are median (IQR; range) or n (%). DA-R-EPOCH=dose-adjusted rituximab, etoposide, prednisolone, vincristine, cyclophosphamide, and doxorubicin. ECOG-PS=Eastern Cooperative Oncology Group Performance Status. HBV=hepatitis B virus. HCV=hepatitis C virus. IPI=International Prognostic Index. LDH=lactate dehydrogenase. R-CHOP=rituximab, cyclophosphamide, doxorubicin, vincristine, and prednisone. R-VACOP=rituximab, etoposide, doxorubicin, cyclophosphamide, vincristine, and prednisone.

* B symptoms are systemic symptoms—ie, fever of unknown origin, night sweats, and weight loss.

† Details on involved extranodal organs are provided in the appendix (p 6).

‡ Median ECOG-PS was 1, with score 0 in 15 (20%) patients, 1 in 32 (43%) patients, 2 in 20 (27%) patients, and 3 in eight (11%) patients.

R-CHOP was the first-line treatment in most of the 43 patients registered at relapse, with a median time to CNS involvement of 5 months (IQR 2–8). Of these 43 patients, 39 (91%) had CNS relapse during the first year of follow-up and 20 (47%) had a systemic lymphoma refractory to the previous line of treatment; refractoriness was defined as progressive disease occurring during treatment or at re-staging after the last course of chemotherapy. 13 (17%) of 75 patients had a previous indolent lymphoma (ten [13%] had follicular lymphoma, one [1%] had lymphoplasmacytic lymphoma, one [1%] had marginal zone lymphoma, and one [1%] had chronic lymphocytic leukaemia); transformation to diffuse large B-cell lymphoma in the CNS was confirmed by brain biopsy in these 13 patients. Characteristics of patients with transformed lymphoma and de-novo diffuse large B-cell lymphoma are shown in the appendix (p 7). The cell of origin was defined according to Hans algorithm; complete data were available for 64 (85%) patients and at least one immunostaining was absent in the other 11 (15%) patients with unclassifiable lymphomas. 38 (59%) of the 64 assessed lymphomas were non-germinal-centre-like subtype.

**Figure 2:**
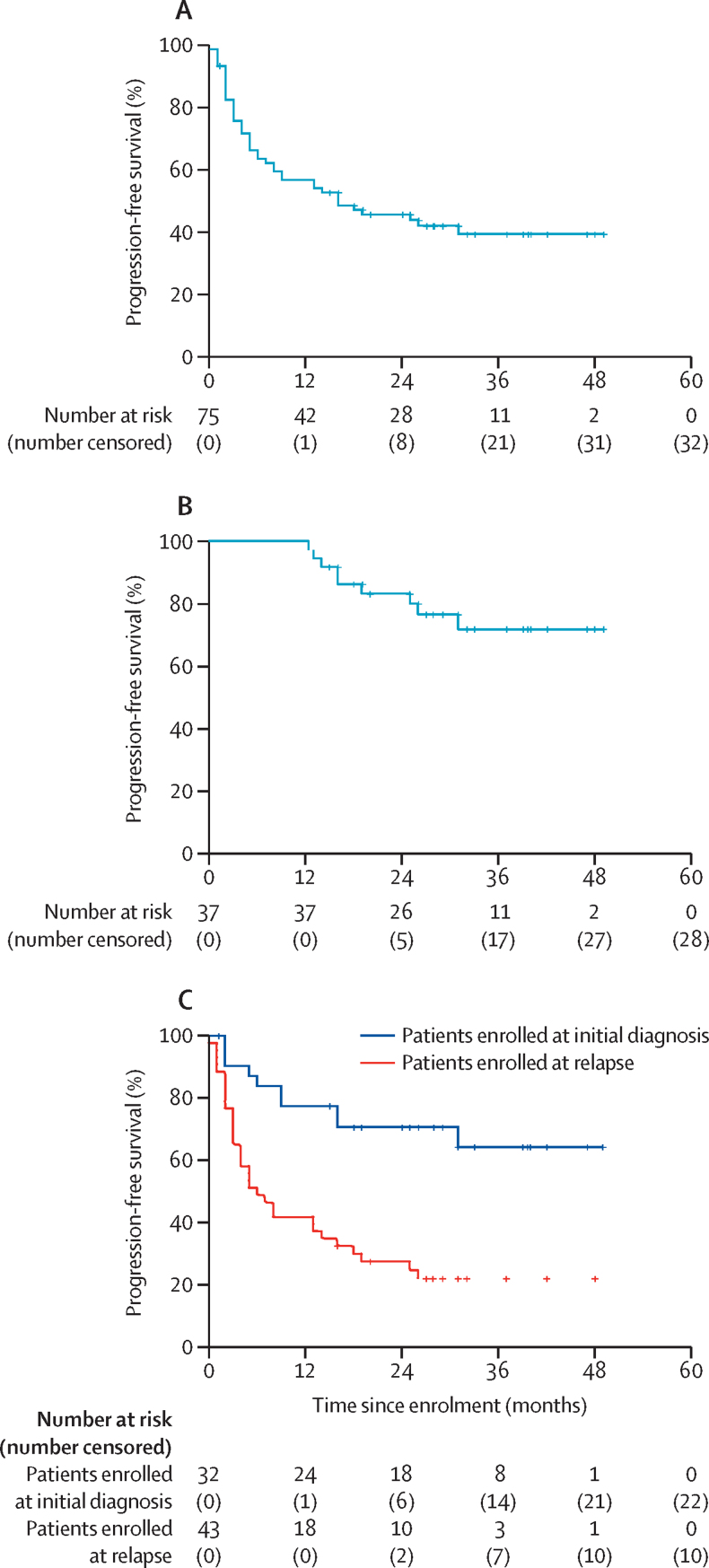
Kaplan-Meier curves of progression-free survival (A) Progression-free survival of the assessable population. (B) Progression-free survival of the 37 transplanted patients. (C) Progression-free survival of the assessable population, according to disease status at trial registration.

At 1 year from enrolment, 42 patients were progression free; with a 1-year progression-free survival of 58% (95% CI 55–61) for the total assessable population and 100% (100–100) for the patients who received a transplant (figure 2A–B). At a median follow-up of 29 months (IQR 20–40), 35 (47%) of the 75 assessable patients were progression free; three of them died without evidence of lymphoma relapse at 13 months (sudden death), 16 months (progressive neurological decline), and 31 months (suspected pulmonary thromboembolism). 25 (33%) of 75 patients had progressive disease during chemotherapy at every step of experimental treatment, 11 (24%) of 46 responders had tumour relapse. Most organs involved at relapse or progression were primary sites of disease (appendix p 9). In exploratory analyses, 2-year progression-free survival was 46% (95% CI 39–53) for the total assessable population and 83% (82–84) for transplanted patients (figure 2A–B). Furthermore, the 30 patients with histologically confirmed CNS disease and the 45 patients assessed with neuroimaging alone had similar progression-free survival at 2 years (43% [95% CI 30–55] *vs* 47% [39–55]; p=0·67).

Progressive and relapsing disease were very aggressive; only seven (19%) of 36 patients received salvage therapy, with no responses, and with a median survival after relapse or progression from MARIETTA treatment of 1 month (IQR 1–3). Therefore, overall survival was similar to progression-free survival in each subset of patients analysed (figure 2; appendix p 14). 33 patients were alive at last follow-up, with a 2-year overall survival of 46% (95% CI 39–53) for the total assessable population and 83% (82–84) for transplanted patients (appendix p 14).

The 75 assessable patients received the first course of MATRix; per protocol, MATRix was preceded by debulking R-CHOP in nine (28%) of the 32 patients enrolled at original lymphoma diagnosis. Post-hoc analysis showed that 55 (73%; 95% CI 63–83) patients had an objective response after two courses of MATRix (table 2). 19 (95%) of the 20 patients who had a complete response after two courses of MATRix maintained the remission after crossing to RICE; 36 patients achieved partial tumour response as the best response during the first two courses of MATRix (one after the first course and 35 after the second one): 28 of them received the third course of MATRix and crossed to RICE, three crossed directly to RICE (protocol violation), one received the third course of MATRix and had progressive disease, and four did not receive other treatment. 24 (77%) of the 31 patients who had RICE after a partial response after two courses of MATRix maintained or improved response after RICE. Three (4%) of 75 patients had stable disease after two courses of MATRix: one received the third course of MATRix and had disease progression, one received the third course of MATRix and crossed to RICE, and one crossed directly to RICE; the last two patients had a major response and received autologous HSCT. 12 (16%) of 75 patients had progressive disease during the first two courses of MATRix (figure 1); per protocol, five (7%) patients crossed to RICE (preceded by a third course of MATRix in one of them), had further progressive disease, and died early; the other seven (11%) patients did not receive other treatment because they had aggressive progressive disease, with fast impairment of neurological and general conditions that impeded any further treatment. There were 19 protocol deviations (figure 1).

**Table 2:** Responses of CNS and systemic disease at different timepoints of protocol treatment

	**Whole series (n=75)**	**CNS disease (n=75)**	**Systemic disease (n=60)**	**CNS disease at presentation (n=32)**	**Relapse; isolated CNS disease (n=15)**	**Relapse; CNS–systemic disease (n=28)**
After second MATRix*						
Complete response	20 (27%)	26 (35%)	26 (43%)	11 (34%)	4 (27%)	5 (18%)
Partial response	36 (48%)	31 (41%)	19 (32%)	17 (53%)	6 (40%)	13 (46%)
Objective response	56 (75%)	57 (76%)	45 (75%)	28 (88%)	10 (67%)	18 (64%)
Stable disease	3 (4%)	6 (8%)	3 (5%)	1 (3%)	1 (7%)	1 (4%)
Progressive disease	12 (16%)	8 (11%)	10 (17%)	3 (9%)	2 (13%)	7 (25%)
**After MATRix–RICE**						
Complete response	29 (39%)	37 (49%)	33 (55%)	17 (53%)	6 (40%)	6 (21%)
Partial response	20 (27%)	14 (19%)	12 (20%)	10 (31%)	3 (20%)	7 (25%)
Objective response	49 (65%)	51 (68%)	45 (75%)	27 (84%)	9 (60%)	13 (46%)
Stable disease	0	0	0	0	0	0
Progressive disease	22 (29%)	20 (27%)	13 (22%)	5 (16%)	4 (27%)	13 (46%)
**Whole treatment**						
Complete response	41 (55%)	44 (59%)	40 (67%)	24 (75%)	7 (47%)	9 (32%)
Partial response	5 (7%)	2 (3%)	4 (7%)	2 (6%)	0	3 (11%)
Objective response	46 (61%)	46 (61%)	44 (73%)	26 (81%)	7 (47%)	12 (43%)
Stable disease	0	0	0	0	0	0
Progressive disease	25 (33%)	25 (33%)	14 (23%)	6 (19%)	6 (40%)	14 (50%)

Data are n (%). Whole series column shows all responses in all patients, CNS disease column shows responses related only to CNS disease (all patients); and systemic disease column shows responses only related to extra-CNS disease in patients who had extra-CNS disease. MATRix=methotrexate, cytarabine, thiotepa, and rituximab. RICE=rituximab, ifosfamide, carboplatin, and etoposide.

* Four patients died of toxicity during MATRix; two of them had concomitant systemic disease.

49 patients (65%; 95% CI 54–76) had an objective response after MATRix–RICE induction: response was complete in 29 (39%; 28–50) patients and partial in 20 (27% [95% CI 17–37]; eight [40%] patients had residual disease in the CNS, six [30%] had residual disease in extra-CNS organs, and six [30%] patients had residual disease in both).

48 (64%) patients were referred for leukapheresis for autologous peripheral blood stem-cell collection (appendix p 8). This procedure was successful (>2 × 10^6^ CD34^+^ cells per kg) in 42 (88%) of the 48 patients, with a median of 6·75 × 10^6^ CD34^+^ cells per kg (IQR 5–8). Autologous peripheral blood stem cells collected after a previous line of treatment and stem cells collected by marrow harvest were used in one patient each.

12 (24%) of the 49 responders were not eligible for autologous HSCT (figure 1); four (33%) of the 12 remained relapse-free at 18–28 months of follow-up. 37 (76%) of the 49 responders (25 complete responses; 12 partial responses) received autologous HSCT. In the 12 patients with partial responses, residual disease was in the CNS in four (33%), in systemic organs in three (25%), and in both in five (42%). Ten (83%) of the 12 patients with partial responses had a complete response after autologous HSCT; three of them received non-protocol planned, post-transplant adjuvant radiotherapy based on the decision of the physician, and the two patients with residual disease after transplantation received radiotherapy (figure 1). Including the two patients who received whole-brain radiotherapy after autologous HSCT, 13 (17%) patients received whole-brain radiotherapy as part of experimental treatment: nine received radiotherapy due to residual (five patients) or progressive (four patients) disease in the brain after or during MATRix–RICE; two patients in complete response after MATRix–RICE received whole-brain radiotherapy because they were poor mobilisers. Seven (78%) of the nine patients who received whole-brain radiotherapy to control responsive disease had a complete or partial response and only one of them had relapsing disease in the brain. None of the four patients who received whole-brain radiotherapy to control progressive disease in the brain responded; all of them died within 9 months of trial enrolment.

46 patients (61%; 95% CI 51–71) had objective evidence of response at initiation of follow-up, with complete response in 41 patients (55%; 44–66). The median duration of response was 26 months (IQR 16–37).

42 (56%) patients died; causes were lymphoma (n=35), toxicity (n=4), progressive neurological decline (n=1), and pulmonary thromboembolism (n=1); cause of death is unknown in one patient (sudden death).

No cases of unexpected toxicity were recorded after debulking R-CHOP. 319 (71%) of the 450 planned MATRix–RICE courses were delivered (appendix p 10); dose reductions were indicated in 32 (10%) courses in 24 (32%) patients. Interruptions due to toxicity occurred only during MATRix: interruption was permanent in four patients and transient in five patients (appendix p 10). 64 (85%) patients received intrathecal chemotherapy. Grade 3–4 toxicity was almost exclusively haematological (table 3): neutropenia in 46 (61%) patients, thrombocytopenia in 45 (60%), and anaemia in 26 (35%). Grade 3 infections occurred in 14 (19%) patients and grade 4 in eight (11%) patients; grade 3 neutropenic fever occurred in ten (13%) patients and grade 4 in one (1%) patient (appendix p 10). 79 serious adverse events (all grades) were recorded in 42 (56%) patients, mostly febrile neutropenia (n=39 episodes) and infections (n=25). Serious adverse events also included bleeding (n=4), bowel perforation (n=2), acute renal failure (n=2), acute neurotoxicity (n=2), pulmonary thromboembolism (n=1), atrial fibrillation (n=1), vomiting plus diarrhoea (n=1), ischaemic stroke (n=1), and increased alanine aminotransferase (n=1). Hospital admission was prolonged in 72 (91%) of 79 serious adverse events; 75 (95%) serious adverse events were followed by recovery, four (5%) were lethal due to sepsis caused by Gram-negative bacteria (*Enterococcous faecalis* and *Escherichia coli*; treatment-related mortality 5%; 95% CI 0·07–9·93). The lethal serious adverse events occurred during MATRix (appendix p 10). There were no differences in toxicity between treatment-naive and previously treated patients: 32 serious adverse events were recorded in 16 (50%) of 32 treatment-naive patients and 47 serious adverse events were recorded in 26 (60%) of 43 previously treated patients (p=0·48); MATRix dose reductions were indicated in nine (28%) of 32 treatment-naive patients and in 15 (35%) of 43 previously treated patients (p=0·53). In the 55 patients in whom RICE was started, RICE dose reductions were indicated in five (19%) of 27 treatment-naive patients and three (11%) of 28 previously treated patients (p=0·77). MATRix was interrupted in three (9%) of 32 treatment-naive patients and six (14%) of 43 previously treated patients (p=0·54). The most common toxicities after autologous HSCT were haematological, followed by mucositis (appendix p 10).

**Table 3:** Adverse events

	**Grade 1–2**	**Grade 3**	**Grade 4**	**Grade 5**
Neutropenia	2 (3%)	5 (7%)	41 (55%)	0
Anaemia	8 (11%)	22 (29%)	4 (5%)	0
Thrombocytopenia	2 (3%)	1 (1%)	44 (59%)	0
Infections	17 (23%)	14 (19%)	8 (11%)	4 (5%)
Hepatotoxicity	8 (11%)	13 (17%)	0	0
Nephrotoxicity	7 (9%)	0	1 (1%)	0
Mucositis	8 (11%)	3 (4%)	1 (1%)	0
Nausea, vomiting, or diarrhoea	17 (23%)	3 (4%)	0	0
Central and peripheral neurotoxicity	10 (13%)	3 (4%)	0	0
Cardiotoxicity	2 (3%)	1 (1%)	0	0
Vascular events*	2 (3%)	1 (1%)	1 (1%)	0

Grade 1 or 2 adverse events are reported if occurring in at least 10% of patients in the treated population (n=75). In patients with multiple concomitant toxicities, each side-effect was considered and reported in the table separately.

* Deep vein thrombosis, pulmonary thromboembolism, or stroke.

Some post-hoc exploratory subgroup analyses were done. Univariate analyses showed that patients with CNS localisation at initial diagnosis had a significantly improved progression-free survival compared with patients with CNS involvement at relapse, with a 2-year progression free survival of 71% (95% CI 69–73) for the 32 patients enrolled at initial lymphoma diagnosis and 28% (11–47) for the 43 patients enrolled at relapse (p=0·0031; figure 2C; appendix p 11). Multivariable analysis showed that CNS involvement at initial lymphoma diagnosis (*vs* CNS involvement at relapse) and complete response to the first two MATRix courses were independently associated with improved progression-free survival (appendix pp 11, 15–16). Notably, the 28 patients enrolled at initial diagnosis with lymphoma responsive to MATRix had a 2-year progression-free survival of 77% (95% CI 76–78; appendix p 17).

Eight (89%) of the nine patients enrolled at initial diagnosis who received debulking R-CHOP before MATRix were relapse-free survivors at 26–47 months of follow-up (appendix p 18). The use of intrathecal chemotherapy was significantly associated with improved progression-free survival (appendix p 19); the 11 patients who did not receive intrathecal chemotherapy had a contraindication to lumbar puncture due to large brain lesions; only one of these patients remained relapse-free at 42 months of follow-up, with a 2-year progression-free survival of 18% (95% CI 0–36) compared with 47% (37–57) for the 64 patients who received intrathecal chemotherapy (p=0·012). The type of intrathecal chemotherapy used (conventional triple drug *vs* liposomal cytarabine) had no effect on progression-free survival (appendix p 19). The history of indolent lymphoma and cell of origin were not associated with outcome (appendix pp 20–21). Age, sex, performance status, and lactate dehydrogenase serum level were not independently associated with outcome (appendix p 11).

## Discussion

To our knowledge, the MARIETTA study is the largest prospective trial focused on patients with secondary CNS lymphoma; it was done in 24 centres in four countries, representing the most geographically extensive trial in patients with secondary CNS lymphoma to date, which supports the generalisability of results. This trial showed that the sequential combination of MATRix and RICE followed by autologous HSCT was active in this population of patients with a very poor prognosis, meeting the predetermined threshold for progression-free survival, without major safety concerns. Response to MATRix was an independent favourable prognostic factor, whereas patients with MATRix-refractory disease had little benefit from crossing to RICE and autologous HSCT. Survival of patients who had a transplantation was encouraging, with significantly improved progression-free survival and overall survival in chemotherapy-naive patients treated at presentation compared with patients who had CNS relapse after a first-line chemoimmunotherapy.

This trial has a few limitations. In particular, the rarity of secondary CNS lymphoma makes it difficult to do randomised trials and only single-arm phase 2 trials seem to be feasible in this patient population. Importantly, the MARIETTA trial considered patients with CNS involvement at both initial diagnosis and relapse, including patients with high-grade transformed diffuse large B-cell lymphoma, and both with isolated CNS relapse and concomitant CNS–systemic disease, which might have generated an interpretation bias related to an apparent study population heterogeneity, reducing the strength of the reported associations. However, patient heterogeneity is a characteristic of this rare lymphoma, and the patients enrolled in this trial reflect the situation in routine practice, with the exception of patients older than 70 years who were excluded; thus, the use of the MARIETTA programme in older patients should be considered with caution. Moreover, studies clearly reporting the ratio between CNS involvement at presentation and at relapse in routine practice do not exist, which does not allow us to put the patient distribution in the present trial in context. However, the relatively large sample size of the MARIETTA trial allowed us to draw reliable conclusions on safety and activity in the whole study population and to distinguish different outcomes between patients with CNS disease at initial lymphoma diagnosis and patients who had CNS dissemination during or after upfront R-CHOP therapy, suggesting that these two secondary CNS lymphoma populations might benefit from different treatments. This finding is an important contribution considering that only one previous study focused on patients with CNS involvement at initial diagnosis of diffuse large B-cell lymphoma exists, which included only 16 patients with this condition.^11^ An additional limitation was that no centralised review of imaging was done, and response and relapse assessment by local investigators of the 24 participating institutions was used as supportive evidence, which might have generated an interpretation bias. However, we can exclude a biased effect on the primary endpoint of the trial because progressive or relapsing disease is a clear condition to treating physicians, usually associated with relevant symptoms and general or neurological impairment. The MARIETTA programme was feasible, with 71% of MATRix–RICE courses delivered, and only four (5%) patients having permanent interruptions (appendix p 10) due to lethal sepsis. Although these rates seem to be a little better than that expected in routine practice, we cannot put these figures in context because retrospective studies on patients treated in everyday practice were not designed to answer this question. Finally, neurotoxicity was an uncommon event in patients treated with the MARIETTA programme; however, potential late cognitive decline cannot be excluded because assessment by neuropsychological tests was not included in the trial design.

When putting the MARIETTA trial in context with previous trials, we note that the study population includes a relatively large proportion of patients with a poor prognosis (appendix p 12): 47% of patients had R-CHOP-refractory lymphoma, 80% of patients had concomitant systemic disease, and 56% had extra-CNS extranodal disease; all these are well known negative prognosticators, which makes it difficult to compare these results with previous trials (three multicentre, single-arm, phase 2 trials done in Germany,^10^ Italy,^11^ and the Netherlands^12^). The only study published at the time of design of the MARIETTA trial was used to estimate the present sample size.^10^ In that trial, a methotrexate–ifosfamide combination followed by cytarabine–thiotepa combination and autologous HSCT had been adopted in 30 patients, reporting a complete response rate after induction of 23%, and a 1-year progression-free survival of 49% for the whole population and 58% for transplanted patients. Comparison between that study and the MARIETTA trial is limited by some relevant differences in selection criteria. In the previous trial,^10^ the upper age limit was 65 years, with an upper limit of ECOG-PS of 2; patients with CNS involvement at initial lymphoma diagnosis were not considered, whereas patients with T-cell lymphomas were also enrolled. The SCNSL1 study^11^ used similar selection criteria to those used in the MARIETTA trial. The characteristics of the 38 patients enrolled in the SCNSL1 trial are similar to those recorded for the present study population (appendix p 12), with the exception of eligible histology (as a few patients with follicular lymphoma or mantle cell lymphoma were enrolled in the SCNSL1 trial) and, importantly, the proportion of patients with disease refractory to the previous line of treatment, which was almost three times greater in the MARIETTA trial.

The chemoimmunotherapy combination assessed in the MARIETTA trial was active in every subgroup of secondary CNS lymphoma, with the best results shown in patients with CNS disease at initial lymphoma diagnosis (43% of the enrolled participants). The SCNSL1 trial has shown a 2-year progression-free survival of 45% in a group of 16 patients with CNS disease at initial diagnosis,^11^ and to our knowledge no other trials have focused on this crucial subgroup of patients. In our study, we report a 2-year progression-free survival of 71% (appendix p 11), an encouraging result considering that the 2-year progression-free survival for patients with diffuse large B-cell lymphoma without CNS involvement treated with R-CHOP is 70–75%.^21^

The safety profile of the MARIETTA programme could be considered to be favourable compared with that reported in the SCNSL1 trial, which consisted of an intensified strategy associated with grade 3–4 infections or febrile neutropenia in 24% of delivered courses (compared with 13% in the MARIETTA trial; appendix p 10) and 11% treatment-related mortality (compared with 5% in the MARIETTA trial). Importantly, intensification high-dose sequential chemoimmunotherapy of the SCNSL1 trial is used only in selected centres in Italy, whereas MATRix and RICE are two standardised regimens that are used in routine practice in several countries. A study from June, 2020,^22^ shows that MATRix is used in routine practice in many cancer centres with similar activity and tolerability to that reported within prospective trials.^13^

In conclusion, the results of the MARIETTA trial are a step forward in the treatment of secondary CNS lymphoma. In particular, progression-free survival for transplanted patients is encouraging and constitutes a good platform to discuss future strategies in this hard-to-treat patient cohort. Patients with CNS involvement at initial diagnosis seem to benefit from treatment with debulking R-CHOP followed by MATRix–RICE and autologous HSCT. Further efforts are urgently required to improve remission before transplantation, especially in patients with CNS involvement at first relapse. Different therapies for patients with CNS involvement at presentation or relapse should be addressed in future trials.

## Data sharing

Participant data is stored on a secure server at the Istituto Oncologico della Svizzera Italiana, Bellinzona, Switzerland, where each participant has been assigned a de-identified trial number. A data dictionary will be available and will include descriptions of patient demographics, treatment, and primary outcome data. Any requests for access to the MARIETTA trial data should be sent to the sponsor (the International Extranodal Lymphoma Study Group; IELSG) and agreement will be made through the data access committee, which will comprise the principal investigators from the trial management group. No identifiable data (ie, name, address, hospital number, NHS number, date of birth, or any other identifying data) will be shared and should not be requested. For each data sharing request, a proforma should be completed, describing the purpose, scope, data items requested, and analysis plan. Requestors who are granted access to the data will be required to complete a data sharing agreement that will be signed by the requester, sponsor, and principal investigator or investigators and should confirm that the trial management team acknowledge the agreement. The study protocol and consent forms are available upon request from IELSG.
