# Supplementary material for: MATRix–RICE therapy and autologous haematopoietic stem-cell transplantation in diffuse large B-cell lymphoma with secondary CNS involvement (MARIETTA): an international, single-arm, phase 2 trial
Source: Lancet Haematol. 2021 Jan 26;8(2):e110–21. doi: 10.1016/S2352-3026(20)30366-5 (PMC7844712; doi:10.1016/S2352-3026(20)30366-5)
Supplement: Supplementary appendix [file mmc1.pdf]

THE LANCET
Haematology

Supplementary appendix

This appendix formed part of the original submission and has been peer reviewed. We post it as supplied by the authors.

Supplement to: Ferreri AJM, Doorduijn JK, Re A, et al. MATRix–RICE therapy and autologous haematopoietic stem-cell transplantation in diffuse large B-cell lymphoma with secondary CNS involvement (MARIETTA): an international, single-arm, phase 2 trial. *Lancet Haematol* 2021; **8:** e110–21.

MATRIX-RICE THERAPY AND AUTOLOGOUS TRANSPLANT IN DIFFUSE LARGE B-CELL LYMPHOMA WITH CNS INVOLVEMENT: A PHASE II TRIAL OF THE INTERNATIONAL EXTRANODAL LYMPHOMA STUDY GROUP

Index	Page
List of participating centers (enrolled patients)	2
Selection of patients (complete list of inclusion and exclusion criteria)	3
Rationale of MARIETTA program	4
Staging work-up and pre-treatment tests	5
Table S1. Extranodal organs involved at initial diagnosis	6
Table S2. Data of patients with high-grade transformation	7
Table S3. Autologous stem cell collection according to chemotherapy course	8
Table S4. Distribution of sites of relapse according to organs involved at trial registration	9
Table S5. Grade ≥3 toxicity reported per course	10
Table S6. Prognostic factors: univariate and multivariable analysis	11
Table S7. Comparison of patient characteristics among MARIETTA study and prior trials on SCNSL patients	12
Figure S1: Flow chart of therapeutic program	13
Figure S2. Overall survival curves of the whole series (A) and the transplanted patients (B)	14
Figure S3. PFS according to lymphoma extension at trial registration	15
Figure S4. PFS curves according to response to the first two MATRIX courses	16
Figure S5. PFS curve of patients registered at initial diagnosis and responsive to MATRIX	17
Figure S6. Effect of debulking R-CHOP on PFS in patients enrolled at presentation	18
Figure S7. Effect of intrathecal chemotherapy on PFS	19
Figure S8. PFS curves of patients with high-grade transformed lymphoma and the others	20
Figure S9. Progression-free survival according to cell of origin	21
References	22
Protocol	23

LIST OF PARTICIPATING CENTERS

Hospital	City, Country	Principal Investigator	Enrolled patients
IRCCS San Raffaele Scientific Institute	Milan, Italy	Andrés J.M. Ferreri	15
Spedali Civili	Brescia, Italy	Giuseppe Rossi	10
UCLH	London, UK	Kate Cwynarski	8
Ospedale Oncologico "Businico"	Cagliari, Italy	Maria G. Cabras	5
Erasmus MC Cancer Institute	Rotterdam, The Nederlands	Jeanette Doorduijn	4
Ospedale della Carità	Novara, Italy	Gianluca Gaidano	4
Aintree	Liverpool, UK	Jeffery Smith	4
Arcispedale Santa Maria Nuova - IRCCS	Reggio Emilia, Italy	Fiorella Ilariucci	3
Policlinico Universitario	Modena, Italy	Franco Narni	3
Beatson Cancer Centre	Glasgow, UK	Pam McKay	3
Villa Sofia – Cervello	Palermo, Italy	Caterina Patti	2
AO Parma	Parma, Italy	Francesca Re	2
Ospedale di Ravenna - IRST	Ravenna, Italy	Claudia Cellini	2
AOU Città della Salute e della Scienza	Turin, Italy	Barbara Botto	2
AOU "Santa Maria della Misericordia"	Udine, Italy	Jacopo Olivieri	2
The Christie	Manchester, UK	Kim Linton	2
Azienda Ospedaliera di Padova	Padua, Italy	Renato Zambello	1
Ospedale di Terni	Terni, Italy	Anna M. Liberati	1
Casa Sollievo della sofferenza	San Giovanni Rotondo, Italy	Nicola Cascavilla	1
San Bortolo ; ULSS n. 6	Vicenza, Italy	Maurizio Frezzato	1
NUTH	Newcastle upon Tyne, UK	Wendy Osborne	1
Nottingham University Hospitals NHS Trust	Nottingham, UK	Christopher Fox	1
Cancer Research UK Centre	Southampton, UK	Andrew Davies	1
Inselspital, Bern University	Bern, Switzerland	Urban Novak	1

SELECTION OF PATIENTS

Inclusion criteria

1. Histologically confirmed diagnosis of diffuse large B-cell lymphoma
2. CNS involvement (brain, meninges, cranial nerves, eyes and/or spinal cord) at diagnosis (concomitant to extra-CNS disease) or relapse after conventional chemo(-immuno)therapy
3. Diagnosis of CNS involvement either by brain biopsy or CSF cytology examination. Neuroimaging alone is acceptable when stereotactic biopsy is formally contraindicated or when the disease has been previously histologically documented in other areas and the CNS localization is clinically defined by fast tumor progression accomplished by consistent neuroimaging findings.
4. No previous treatment with high-dose methotrexate-based chemotherapy and/or brain irradiation.
5. Age 18-70 years
6. ECOG performance status 0-3
7. Adequate bone marrow (Platelets count $\geq$100 x10^3/mm^3, hemoglobin $\geq$9 g/dL, neutrophils count $\geq$1.5 x 10^3/mm^3), renal (creatinine clearance $\geq$60 mL/min), cardiac (LVEF $\geq$50%), and hepatic function (total serum bilirubine $\leq$3 mg/dL, AST/ALT and GGT $\leq$2.5 per upper normal limit value), unless the abnormality is due to lymphoma infiltration
8. Absence of HIV infection and of detectable HCV-RNA and/or HBsAg and/or HBV-DNA
9. No concurrent malignancies. Previous malignancies are accepted if surgically cured or if there was no evidence of disease in the last 3 years at a regular follow-up
10. Absence of any familial, sociological or geographical condition potentially hampering compliance with the study protocol and follow-up schedule
11. Female patients must be non-pregnant and non-lactating. Sexually active patients of childbearing potential must implement adequate contraceptive measures during study participation. Highly effective contraceptive precautions are required until 12 months after the completion of trial treatment. Pregnancy test must be performed within 7 days of commencing study treatment.
12. No treatment with other experimental drugs within the 6 weeks previous to enrolment
13. Given written informed consent prior to any study specific procedures, with the understanding that the patient has the right to withdraw from the study at any time, without any prejudice. Informed consent signed by a patient's guardian is acceptable if the patient is not able to decide inclusion in the study due to cognitive impairment

Exclusion criteria

1. Other lymphoma categories other than diffuse large B-cell lymphoma. In particular, patients with primary mediastinal lymphoma, intravascular large B-cell lymphoma or leg-type large B-cell lymphoma are excluded.
2. Patients with positive flow cytometry examination of the CSF, but negative results in CSF conventional cytology, and without any other evidence of CNS disease.
3. Patients with exclusive CNS disease at presentation (primary CNS lymphoma) are excluded.
4. Previous treatment with support of autologous or allogeneic stem cells/bone marrow transplantation.
5. Symptomatic coronary artery disease, cardiac arrhythmias not well controlled with medication or myocardial infarction within the last 6 months (New York Heart Association Class III or IV heart disease).
6. Any other serious medical condition that could impair the ability of the patient to participate in the trial.
7. Any contraindications as identified in relation to the relevant SmPCs.

RATIONALE OF MARIETTA PROGRAM

- "MARIETTA" is based on two important anti-lymphoma chemotherapy principles: the positive impact of dose intensity/density and the combination of cytostatics active in different cell-cycle phases.
- Anti-cancer agents were selected for both their well-documented anti-lymphoma activity as well as their increased blood-brain barrier (BBB) penetration and CNS bioavailability.
- MARIETTA regimen is based on positive experiences in treating primary CNS lymphoma with high doses of antimetabolites[1], MATRIX in particular[2], and consolidation with BCNU-thiotepa-conditioned autologous stem cell transplantation (ASCT)[3].
- Sequential administration of RICE is based on its well-documented efficacy in patients with relapsed/refractory DLBCL[4], the efficacy of high-dose-ifosfamide-based regimens in patients with primary CNS lymphoma[5], and the lack of cross-resistance with antimetabolites.
- Intrathecal chemotherapy was indicated in every enrolled patient regardless of CSF cytology status because conventional cytology examination is associated with high false negative results; in particular, detection sensitivity of CSF cytology exam is significantly lower when routinely used without flow cytometry[6].
- The MARIETTA regimen includes the latest version thiotepa-BCNU conditioning regimen, with higher doses of thiotepa (20 mg/kg), with similar tolerability and improved efficacy[3].
- Similarly to the SCNSL1 trial[7], the MARIETTA regimen offered the possibility of starting with R-CHOP regimen, in the case of extensive and life-threatening extra-CNS disease, and to deliver whole-brain irradiation (WBRT) in patients with residual CNS disease after ASCT.
- The protocol mandated that patients with progressive disease in the brain parenchyma during MATRix would immediately switch to the RICE regimen, with subsequent CNS progression events treated with WBRT prior to proceeding with ASCT consolidation. This approach is based on retrospective studies demonstrating that patients with SCNSL who achieved CNS disease remission after primary WBRT achieved long-lasting disease control with consolidative ASCT[8].

STAGING WORK-UP AND PRE-TREATMENT TESTS

- Physical and neurological examination
- ECOG performance status definition
- Peripheral blood examination
- Viral markers (HIV, HBV, HCV, CMV, HHV6-8, and parvovirus)
- Echocardiography with determination of LVEF
- Respiratory volumes assessment with diffusion capacity measurements
- Contrast-enhanced neck, thorax and abdomen CT scan
- Ultrasonography of testes in men older than 60 years (or suspected testicular involvement)
- Bone marrow aspirate and/or biopsy
- Gadolinium-enhanced whole-brain MRI
- Cerebrospinal fluid examination (cytology, physicochemical characterization, flow cytometry)
- Ophthalmologic evaluation
- ^{18}FDG-PET

Table S1. Extranodal organs involved at initial diagnosis

Site	Number of patients (n= 75)
Skeleton (every bone other than skull)	16 (21%)
Bone marrow	15 (20%)
Testis	12 (16%)
Base of the skull (including rhinopharynx, paranasal sinuses, orbit)	9 (12%)
Liver	8 (11%)
Lung	7 (9%)
Kidney	6 (8%)
Breast	4 (5%)
Stomach	4 (5%)
Pancreas	4 (5%)
Adrenal gland	4 (5%)
Skin	2 (3%)
Peripheral nerves	2 (3%)
Muscle	1 (1%)
Ovary	1 (1%)
Bowel	1 (1%)
Thyroid gland	1 (1%)
Parotid gland	1 (1%)
Uterus	1 (1%)

Table S2. Data of patients with high-grade transformation

Patients with high-grade transformed lymphoma	13
Age, median (range)	60 (34 – 70)
Male gender	5 (38%)
Disease at trial registration	
CNS involvement at presentation	4 (31%)
Isolated CNS relapse	5 (38%)
Concomitant CNS-systemic localisation	4 (31%)
HBV or HCV seropositivity	1 (8%)
B Symptoms	2 (15%)
CNS sites of disease	
Brain parenchyma	6 (46%)
CSF/meninges	2 (15%)
Brain and CSF/meninges	3 (23%)
Brain and eyes	1 (8%)
Brain, CSF and eyes	1 (8%)
Extra-CNS sites of disease at registration (n= 8)	
Nodal disease (only lymphadenopathies)	2 (25%)
Extranodal disease (other than CNS)	3 (38%)
Nodal and extranodal disease	3 (38%)
International Prognostic Index (IPI)	
Age >60 years old	6 (46%)
ECOG - PS >1	5 (38%)
Extranodal >1 (other than CNS)	4 (31%)
High LDH serum level	6 (46%)
Advanced stage	8 (62%)
Low IPI risk	3 (23%)
Low-intermediate IPI risk	2 (15%)
High-intermediate IPI risk	5 (38%)
High IPI risk	3 (23%)
Prior treatment for DLBCL (n= 9)	
Rituximab-CHOP	8 (89%)
Rituximab-bendamustine	1 (8%)
Prior CNS prophylaxis (only intrathecal chemo)	1 (8%)
Refractory to prior treatment (n= 9)	4 (44%)

Thirteen patients had a prior indolent lymphoma (high-grade transformation before trial registration): follicular lymphoma in 10 patients, lymphoplasmacytic lymphoma, marginal zone lymphoma and chronic lymphocytic leukemia in one patient each; transformation to DLBCL in the CNS was confirmed by brain biopsy in the 13 patients.

Table S3. Autologous stem cell collection according to chemotherapy course

APBSC collected after	Patients referred for leukapheresis	Successful APBSC collection (>2 x 10^6 CD34+ cells/kg)
Second course of MATRIX	25	22 (88%)
Third course of MATRIX	16	15 (94%)
First or second course of RICE	7	5 (72%)
Total	48	42 (88%)

APBSC= autologous peripheral blood stem cell

Table S4. Distribution of sites of relapse according to organs involved at trial registration

Systemic disease at registration	Events[#]	CNS site at event	Systemic site at event	Both CNS and systemic sites at event
YES, at initial diagnosis<br>(n= 32)	8	Brain (5)	Soft tissues (SS; 1)	Brain + multiple organs (1)<br>Brain (SS) + cervical nodes (1)*
YES, at relapse<br>(n= 28)	21	Brain (2)<br>Meninges (2)*<br>Meninges, eyes (SS) (1)*	Multiple sites (1)<br>Abdominal nodes (1)*<br>Multiple organs (SS; 2)<br>Sacrum (SS; 1)	Brain + multiple organs (5)<br>Brain + multiple organs (SS) (4)*<br>Eyes + multiple organs (1)<br>Meninges + multiple organs (1)
NO, at relapse<br>(n= 15)	7	Brain (4)*	Multiple organs (SS; 1)*	Brain + multiple organs (SS) (1)<br>Brain (SS), meninges + multiple organs (SS) (1)

[#] Patients experiencing relapse/progression during/after MARIETTA. Unrelated and toxic deaths are excluded.
Most sites involved at relapse or progression were primary sites, that is organs involved at trial registration; exceptions, called “secondary sites”, that is organs uninvolved at trial registration, were pointed out as “SS”.
Asterisks indicate site of relapse in patients who achieved complete remission after the experimental treatment (n= 7).

Table S5. Grade ≥3 toxicity reported per course

	1st MATRIX	2nd MATRIX	3rd MATRIX	1st RICE	2nd RICE	3rd RICE	ASCT
Number of courses	75	66	48	55	43	32	37
G3 Neutropenia	6 (8%)	1 (2%)	4 (8%)	4 (7%)	3 (7%)	1 (3%)	2 (5%)
G4 neutropenia	28 (37%)	25 (38%)	19 (40%)	17 (31%)	12 (28%)	12 (38%)	35 (95%)
G3 anaemia	6 (8%)	12 (18%)	10 (21%)	12 (22%)	2 (5%)	3 (9%)	3 (8%)
G4 anaemia	0 (0%)	2 (3%)	1 (2%)	1 (2%)	0 (0%)	0 (0%)	5 (14%)
G3 thrombocytopenia	4 (5%)	6 (9%)	2 (4%)	6 (11%)	7 (16%)	1 (3%)	3 (8%)
G4 thrombocytopenia	32 (43%)	27 (41%)	22 (46%)	15 (23%)	11 (26%)	11 (34%)	34 (92%)
G3 neutropenic fever	4 (5%)	1 (2%)	2 (4%)	2 (4%)	1 (2%)	0 (0%)	3 (8%)
G4 neutropenic fever	1 (1%)	0 (0%)	0 (0%)	0 (0%)	0 (0%)	0 (0%)	1 (3%)
G3 infections	7 (9%)	4 (6%)	5 (10%)	5 (9%)	2 (5%)	1 (3%)	4 (11%)
G4 infections	6 (8%)	1 (2%)	1 (2%)	0 (0%)	0 (0%)	0 (0%)	0 (0%)
G3 hepatotoxicity	10 (13%)	3 (5%)	2 (4%)	1 (2%)	0 (0%)	0 (0%)	0 (0%)
G4 hepatotoxicity	0 (0%)	0 (0%)	0 (0%)	0 (0%)	0 (0%)	0 (0%)	0 (0%)
G3 nephrotoxicity	0 (0%)	0 (0%)	0 (0%)	0 (0%)	0 (0%)	0 (0%)	0 (0%)
G4 nephrotoxicity	0 (0%)	0 (0%)	1 (2%)	0 (0%)	0 (0%)	0 (0%)	0 (0%)
G3 mucositis	0 (0%)	0 (0%)	3 (6%)	0 (0%)	0 (0%)	0 (0%)	8 (22%)
G4 mucositis	0 (0%)	1 (2%)	0 (0%)	0 (0%)	0 (0%)	0 (0%)	0 (0%)
G3 N/V/D	0 (0%)	0 (0%)	0 (0%)	2 (4%)	0 (0%)	1 (3%)	1 (3%)
G4 N/V/D	0 (0%)	0 (0%)	0 (0%)	0 (0%)	0 (0%)	0 (0%)	0 (0%)
G3 neurotoxicity	0 (0%)	0 (0%)	0 (0%)	2 (4%)	1 (2%)	0 (0%)	0 (0%)
G4 neurotoxicity	0 (0%)	0 (0%)	0 (0%)	0 (0%)	0 (0%)	0 (0%)	0 (0%)
G3 cardiotoxicity	0 (0%)	1 (2%)	1 (2%)	1 (2%)	0 (0%)	0 (0%)	0 (0%)
G4 cardiotoxicity	0 (0%)	0 (0%)	0 (0%)	0 (0%)	0 (0%)	0 (0%)	0 (0%)
G3 vascular events	0 (0%)	0 (0%)	0 (0%)	1 (2%)	0 (0%)	0 (0%)	0 (0%)
G4 vascular events	1 (1%)	0 (0%)	0 (0%)	0 (0%)	0 (0%)	0 (0%)	0 (0%)
Dose reductions	6 (8%)	9 (14%)	9 (19%)	2 (4%)	4 (9%)	2 (6%)	1 (3%)
Temporary interruptions	4 (5%)	0 (0%)	1 (2%)	0 (0%)	0 (0%)	0 (0%)	0 (0%)
Permanent interruptions	1 (1%)	1 (2%)	2 (4%)	0 (0%)	0 (0%)	0 (0%)	0 (0%)
Toxic deaths	3 (4%)	1 (2%)	0 (0%)	0 (0%)	0 (0%)	0 (0%)	0 (0%)

In patients with multiple concomitant toxicities, each side effect was considered and reported in the table separately.
ASCT= autologous stem cell transplantation.

Table S6. Prognostic factors: univariate and multivariable analysis

Variable	Subgroups	N°	2-year PFS	p value#	HR (95%IC)	p value§
**Age**	Continuous variable	75			1·01 (0·98 – 1·03)	0·418
**Gender**	Female<br>Male	37<br>38	35 ± 8%<br>56 ± 8%	0·038	1<br>0·58 (0·30 – 1·10)	0·096
**ECOG-PS score**	0 - 1<br>2 - 3	47<br>28	55 ± 7%<br>29 ± 9%	0·021	1<br>1·80 (0·93 – 3·46)	0·076
**LDH serum level**	Normal<br>Increased	38<br>37	48 ± 8%<br>43 ± 8%	0·376	-	-
**CNS involvement**	At presentation<br>At relapse	32<br>43	71 ± 8%<br>28 ± 7%	0·003	1<br>2·41 (1·11 – 5·26)	0·025
**CSF/meningeal disease**	No<br>Yes	46<br>29	54 ± 7%<br>31 ± 9%	0·031	1<br>1·96 (0·863 – 4·42)	0·105
**Extranodal organs**	0 – 1<br>2 – 3	39<br>36	51 ± 8%<br>39 ± 8%	0·378	-	-
**Response to MATRIX**[¥]	Complete remission<br>Partial response<br>No response*	20<br>35<br>20	79 ± 9%<br>44 ± 8%<br>10 ± 6%	 0·025<br>0·00001	1<br>0·610 (0·22 – 1·66)<br>17 (5 – 50)	 0·339<br>0·000001

Time zero of the covariates was baseline (at trial registration), with the single exception of “response to the first two MATRIX courses” (¥).Time at risk for the analysis of the prognostic role of response to the first two MATRIX courses started at the date of response assessment.

[#] Univariate analysis; log-rank test.

[§] Multivariate analysis; Cox proportional hazard model.

[¶] Patients registered at first relapse with disease involving both the CNS and systemic organs.

* Patients with progressive or stable disease and patients died of toxicity are included.

N°= number of assessed patients; ECOG-PS= Eastern Cooperative Oncology Group - performance status; LDH= lactic dehydrogenase; CNS= central nervous system; CSF= cerebrospinal fluid.

Table S7. Comparison of patient characteristics among MARIETTA study and prior trials on SCNSL patients

Trial	NCT01148173	SCNSL1	HOVON	MARIETTA
Countries	Germany	Italy	Netherlands	IT, UK, NL, CH
Enrolled patients	33	40	36	78
Eligible patients	30	38	36	75
DLBCL	27	32	35	75
Age, median (range)	58 (29 – 65)	59 (36 – 70)	57 (23 – 65)	58 (23 – 70)
Male gender	15 (50%)	23 (61%)	19 (53%)	38 (51%)
ECOG - PS >2	0 (0%)	6 (16%)	0 (0%)	8 (11%)
High LDH serum level	10 (33%)	21 (55%)	10 (28%)	37 (49%)
Advanced stage	20 (66%)	23 (61%)	NR	60 (80%)
Extranodal disease (other than CNS)	17 (57%)	18 (47%)	NR	38 (51%)
HBV or HCV positivity	0 (0%)	0 (0%)	NR	2 (3%)
International Prognostic Index (IPI)				
0 - 1	8 (27%)	12 (32%)	NR	14 (19%)
2 - 3	17 (57%)	21 (55%)	NR	45 (60%)
4 - 5	4 (13%)	5 (13%)	NR	16 (21%)
Unknown	1 (3%)	0 (0%)	NR	0 (0%)
Disease at trial registration				
At initial lymphoma diagnosis	0 (0%)	16 (42%)	0 (0%)	32 (43%)
Isolated CNS relapse	24 (80%)	15 (39%)	16 (44%)	15 (20%)
Concomitant CNS-systemic relapse	6 (20%)	7 (18%)	20 (56%)	28 (37%)
Prior treatment	30 (100%)	22 (58%)	35 (100%)	43 (57%)
Rituximab-CHOP-like	26 (87%)	20 (91%)	NR	40 (93%)
CHOP or CHOP-like	3 (10%)	1 (3%)	NR	2 (5%)
Others	1 (3%)	1 (3%)	NR	1 (2%)
Prior CNS prophylaxis (intrathecal chemo)	6 (20%)*	4 (11%)	NR	7 (16%)
Refractory to prior treatment (n= 43)	0 (0%)	7 (18%)	NR	20 (47%)

DLBCL= diffuse large B-cell lymphoma; IT= Italy; UK= United Kingdom; NL= The Netherlands; CH= Switzerland.
* One patients received HD-MTX as part of prior treatment.

Figure S1: Flow chart of therapeutic program

IT-CHT= intrathecal chemotherapy; CR= complete remission; PR= partial response; SD= stable disease; PD= progressive disease; HDC/ASCT= high-dose chemotherapy supported by autologous stem-cell transplantation; WBRT= whole-brain radiation therapy.

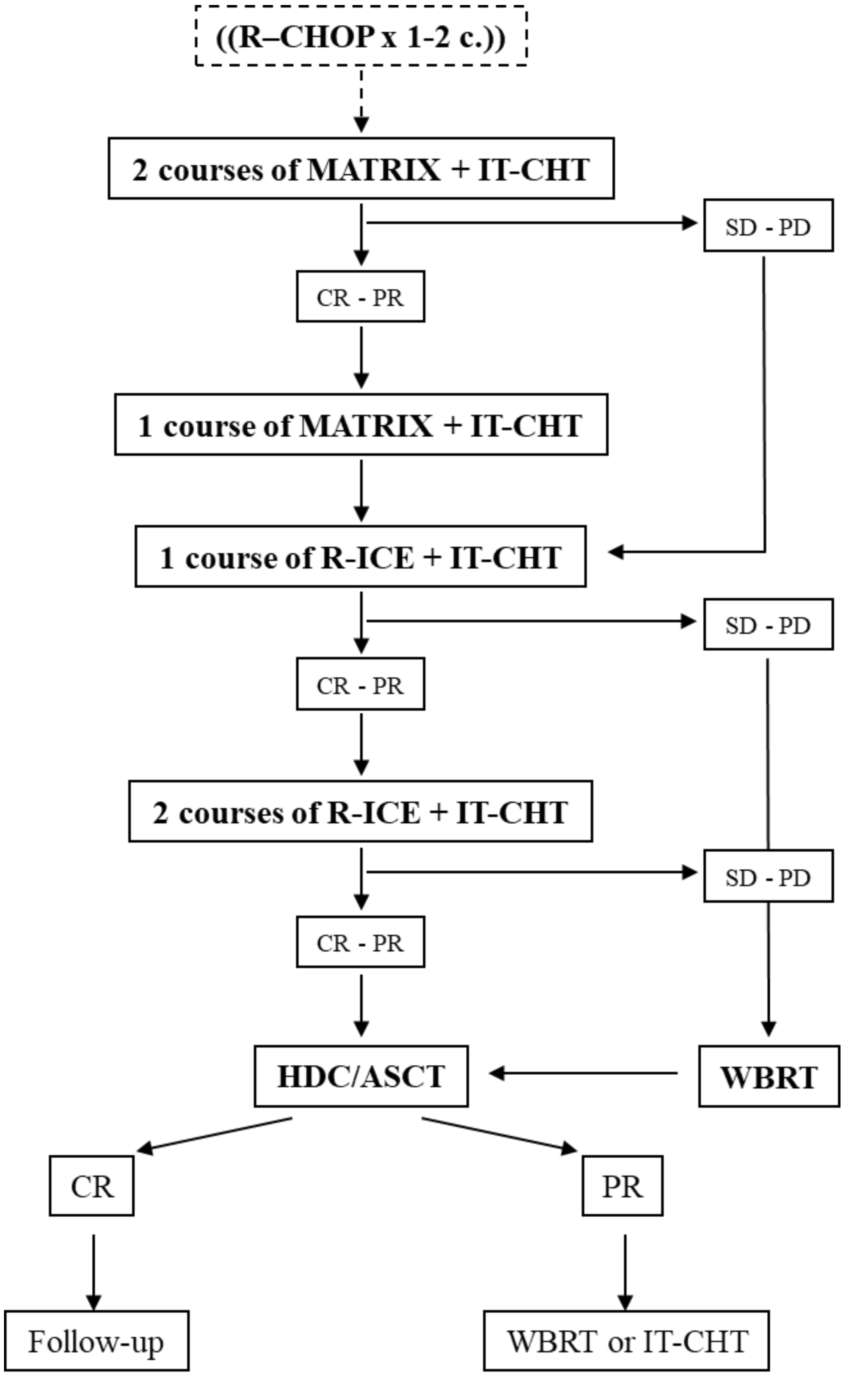

Figure S2. Overall survival curves of the whole series (A) and the transplanted patients (B)

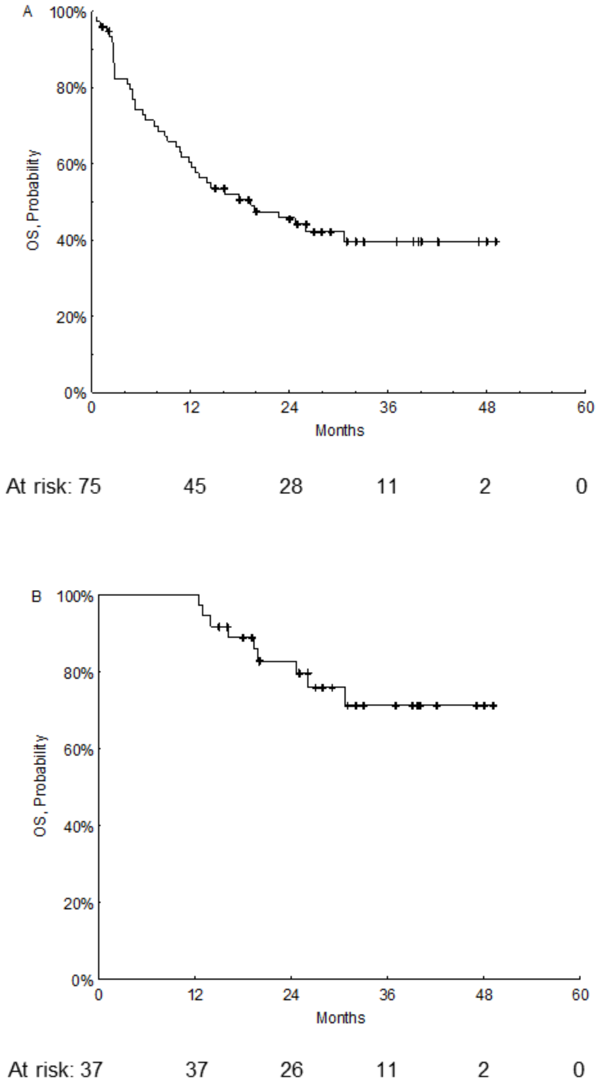

Figure S3. PFS according to lymphoma extension at trial registration

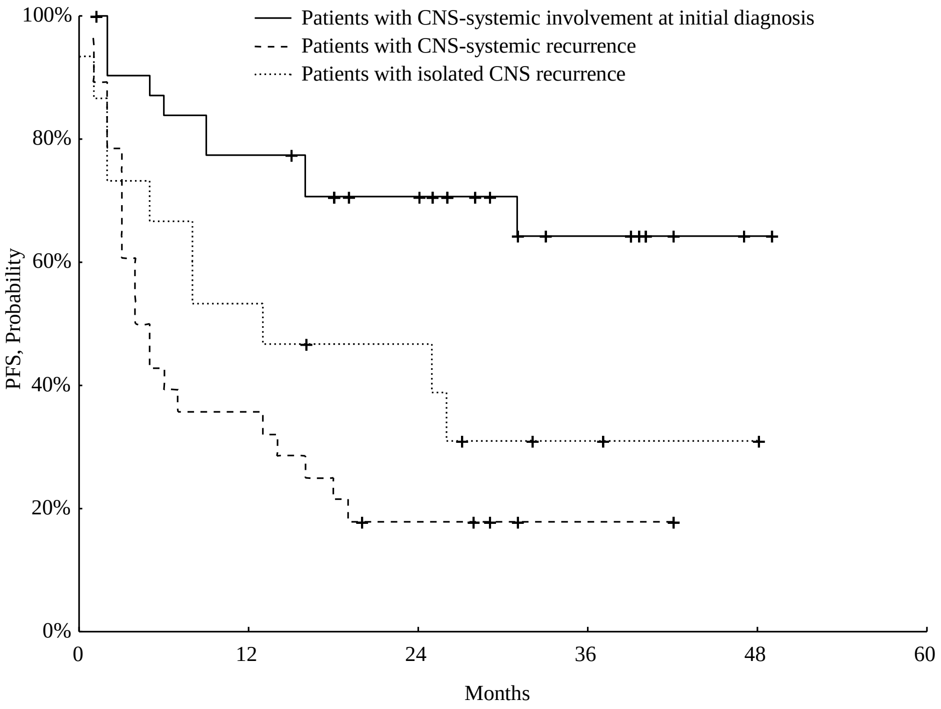

—— At risk: 32	24	18	8	1	0
- - - - At risk: 28	10	4	1	0	0
······ At risk: 15	8	6	2	1	0

Figure S4. PFS curves of the study population divided according to response to the first two MATRIX courses

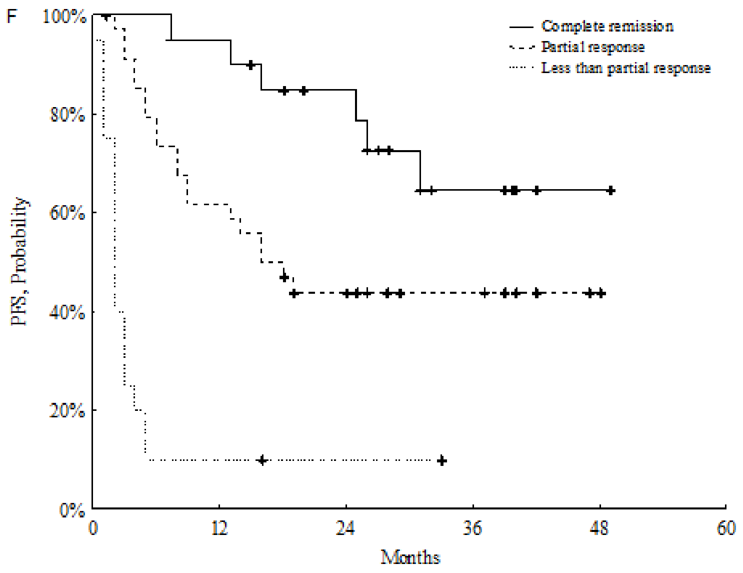

—— At risk:	20	18	13	5	1	0
- - - At risk:	35	20	10	5	1	0
········ At risk:	20	2	1	0	0	0

Figure S5. PFS curve of the 28 patients registered at initial diagnosis with lymphoma responsive to the first two MATRIX courses

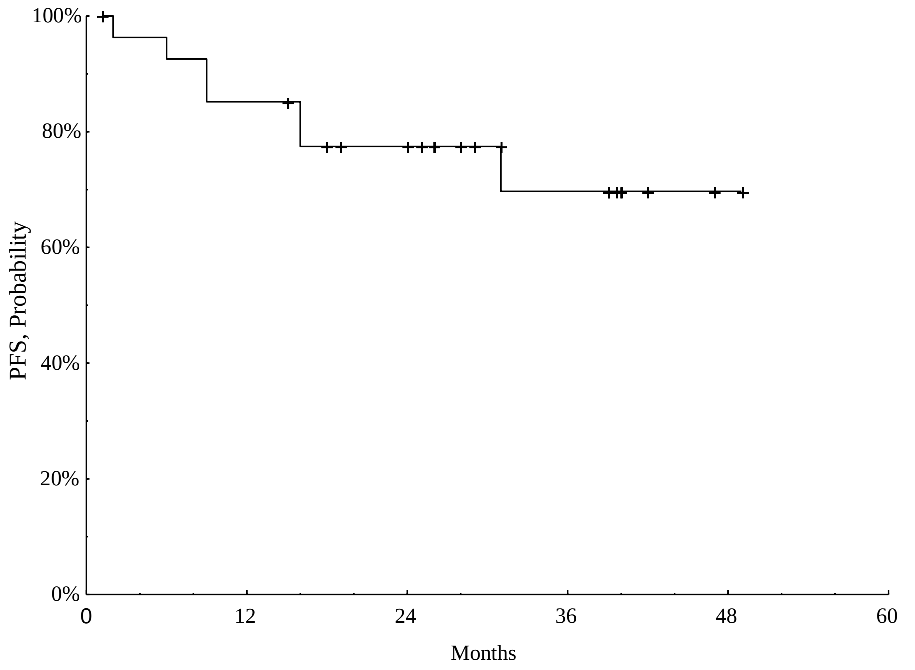

At risk: 28 23 17 8 1 0

Figure S6. Effect of debulkying R-CHOP on PFS in patients enrolled at presentation.

Only one of the nine patients enrolled at initial lymphoma diagnosis and treated with debulkying R-CHOP before MATRIX experienced relapse and died of lymphoma, with a 2-year PFS of 89 ± 10% (continued line) in comparison with the 63 ± 10% for the 23 patients enrolled at initial lymphoma diagnosis and treated without debulkying R-CHOP (dotted line).

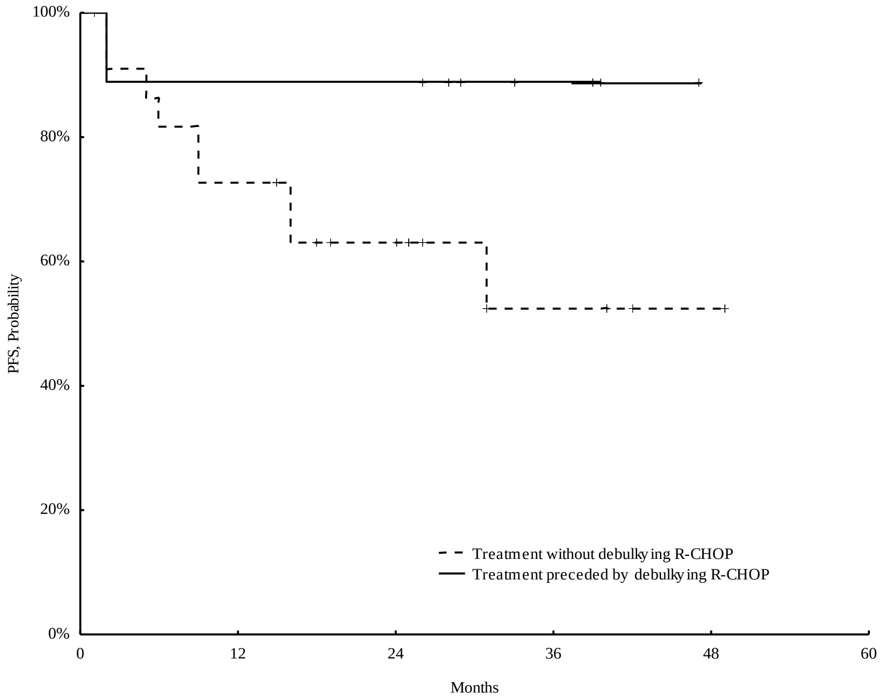

— At risk:	9	8	8	4	0	0
---- At risk:	23	16	10	4	1	0

Figure S7. Effect of intrathecal chemotherapy on PFS.

Patients with contraindication to intrathecal chemotherapy showed a significantly poorer PFS than patients who received this therapy. Only one patient with lumbar puncture contraindication remained relapse-free at 42 months of follow-up, with a 2 year PFS of 18 ± 12% in comparison with 47 ± 9% for the 64 patients who receive intrathecal chemotherapy. Within the later subgroup, the type of used intrathecal chemotherapy (conventional triple drug versus liposomal cytarabine) had no effect on PFS.

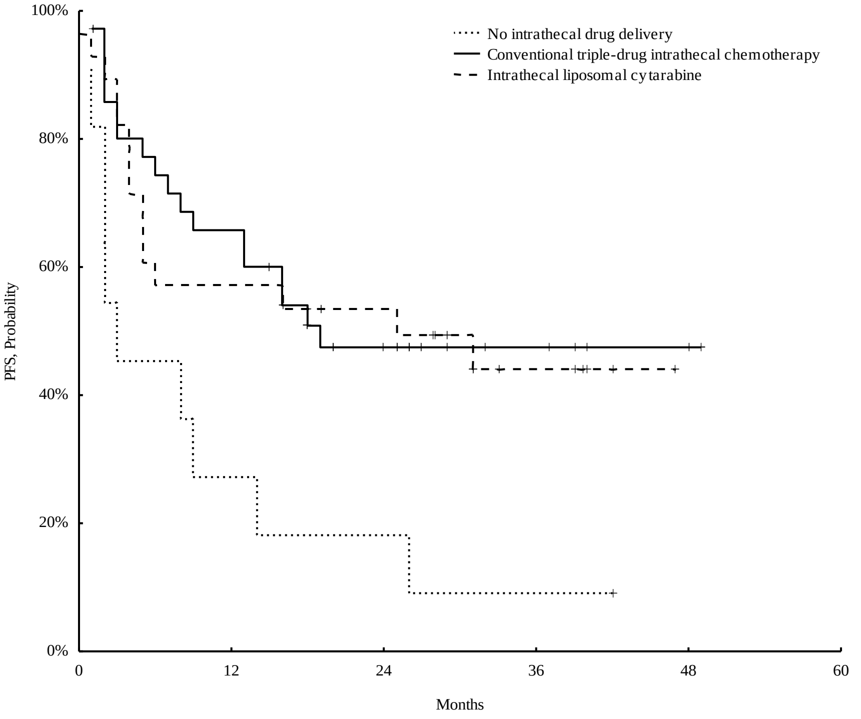

—— At risk:	36	23	13	5	2	0
- - - - At risk:	28	16	13	5	0	0
······· At risk:	11	4	2	1	0	0

Figure S8. Progression-free survival curves of patients with high-grade transformed lymphoma and the others

The 2-year PFS was 37 ± 14% for the 13 patients with high-grade transformed lymphoma (dotted line) and 47 ± 6% for the 60 patients with *de novo* DLBCL (continued line) (p=0.29).

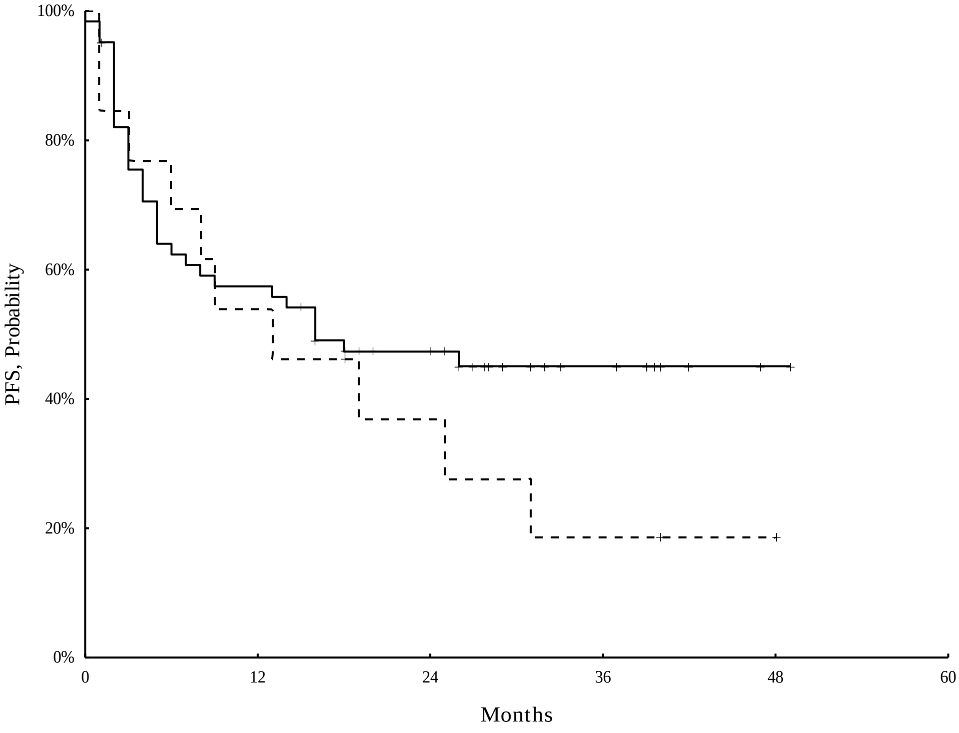

—— At risk:	62	35	24	9	1	0
- - - - At risk:	13	7	4	2	1	0

Figure S9. Progression-free survival according to cell of origin (Hans algorithm)

Cell of origin was not associated with outcome, with a 2-year PFS of 49 ± 10% for germinal-center-like subtype and 50 ± 8% for non-germinal-center-like subtype; p= 0.99).

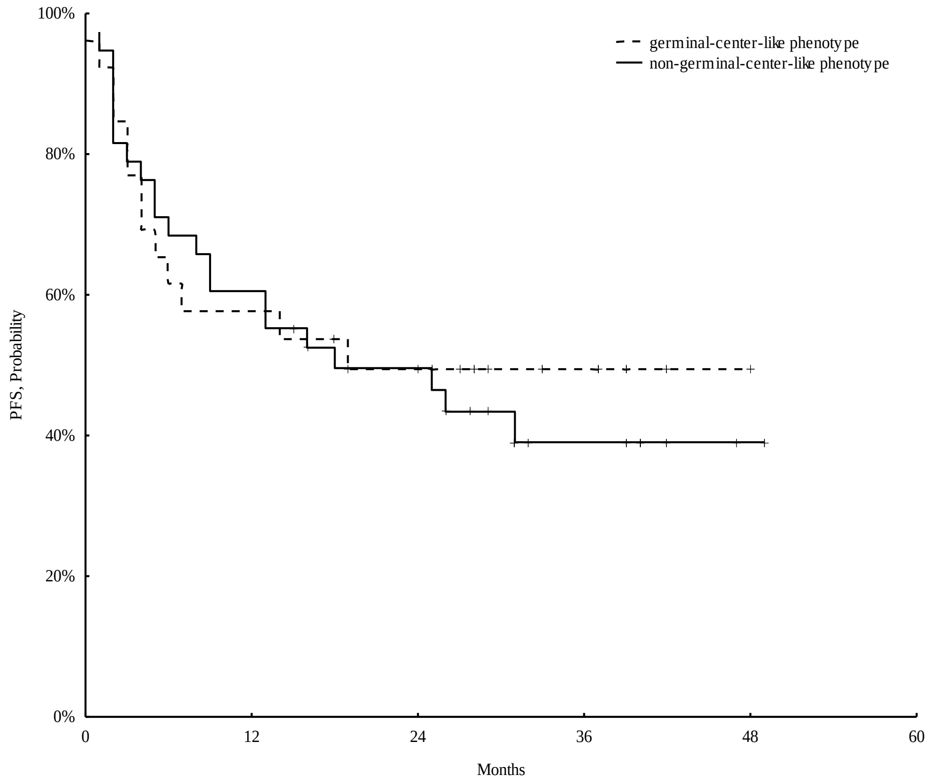

—— At risk:	38	23	17	6	1	0
- - - - At risk:	26	15	10	4	1	0

References

1. Ferreri AJ, Reni M, Foppoli M, et al. High-dose cytarabine plus high-dose methotrexate versus high-dose methotrexate alone in patients with primary CNS lymphoma: a randomised phase 2 trial. *Lancet* 2009; **374**: 1512-20.

2. Ferreri AJ, Cwynarski K, Pulczynski E, et al. Chemoimmunotherapy with methotrexate, cytarabine, thiotepa, and rituximab (MATRix regimen) in patients with primary CNS lymphoma: results of the first randomisation of the International Extranodal Lymphoma Study Group-32 (IELSG32) phase 2 trial. *Lancet Haematol* 2016; **3**(5): e217-27.

3. Kasenda B, Schorb E, Fritsch K, Finke J, Illerhaus G. Prognosis after high-dose chemotherapy followed by autologous stem-cell transplantation as first-line treatment in primary CNS lymphoma--a long-term follow-up study. *Ann Oncol* 2012; **23**(10): 2670-5.

4. Gisselbrecht C, Glass B, Mounier N, et al. Salvage regimens with autologous transplantation for relapsed large B-cell lymphoma in the rituximab era. *J Clin Oncol* 2010; **28**(27): 4184-90.

5. Mappa S, Marturano E, Licata G, et al. Salvage chemoimmunotherapy with rituximab, ifosfamide and etoposide (R-IE regimen) in patients with primary CNS lymphoma relapsed or refractory to high-dose methotrexate-based chemotherapy. *Hematol Oncol* 2012;31(3):143-50.

6. Wilson WH, Bromberg JE, Stetler-Stevenson M, et al. Detection and outcome of occult leptomeningeal disease in diffuse large B-cell lymphoma and Burkitt lymphoma. *Haematologica* 2014; **99**(7): 1228-35.

7. Ferreri AJ, Donadoni G, Cabras MG, et al. High Doses of Antimetabolites Followed by High-Dose Sequential Chemoimmunotherapy and Autologous Stem-Cell Transplantation in Patients With Systemic B-Cell Lymphoma and Secondary CNS Involvement: Final Results of a Multicenter Phase II Trial. *J Clin Oncol* 2015; **33**(33): 3903-10.

8. Williams CD, Pearce R, Taghipour G, Green ES, Philip T, Goldstone AH. Autologous bone marrow transplantation for patients with non-Hodgkin's lymphoma and CNS involvement: those transplanted with active CNS disease have a poor outcome--a report by the European Bone Marrow Transplant Lymphoma Registry. *J Clin Oncol* 1994; **12**(11): 2415-22.

An international phase II trial assessing tolerability and efficacy of sequential Methotrexate-Aracytin-based combination and R-ICE combination, followed by high-dose chemotherapy supported by autologous stem cell transplant, in patients with systemic B-cell lymphoma with central nervous system involvement at diagnosis or relapse (MARIETTA regimen)

EudraCT number **2014-003031-19**

Steering committee

Andrés J. M. Ferreri e-mail ferreri.andres@hsr.it
Chairman
Unit of Lymphoid Malignancies
Department of Oncology
San Raffaele Scientific Institute
20132 Milano, Italy

Emanuele Zucca e-mail emanuele.zucca@eoc.ch
Co-Chair
Oncology Institute of Southern Switzerland
Ospedale San Giovanni
6500 Bellinzona, Switzerland

Kate Cwynarski e-mail kate.cwynarski@uclh.nhs.uk
Co-Chair
Department of Haematology
University College London Hospital, London NW1 2PG

Gerald Illerhaus e-mail g.illerhaus@klinikum-stuttgart.de
Co-Chair
Clinic of Haematology, Oncology and Palliative Medicine
Stuttgart Cancer Center/Oncology Center Eva Mayr-Stihl
70174 Stuttgart, Germany

Armando López-Guillermo e-mail ALOPEZG@clinic.ub.es
Co-Chair
Department of Hematology
Hospital Clinic,
08036 Barcelona, Spain

Tali Siegal/Osnat Bairey e-mail talisi1@clalit.org.il
osnatb@clalit.org.il
Co-Chair
Rabin Medicl Center
Beilinson Hospital
49100 Petach Tikwa, Israel

Jacoline E. C. Bromberg e-mail j.bromberg@erasmusmc.nl
Co-Chair
Department of Neuro-Oncology
Erasmus MC Cancer institute
3000CA Rotterdam, The Netherlands

Jeanette Doorduijn e-mail j.doorduijn@erasmusmc.nl
Co-Chair
Department. of Hematology
Erasmus MC Cancer Institute
3000CA Rotterdam, The Netherlands

Study Coordination

IELSG Coordinating Centre
Oncology Institute of Southern Switzerland
Ospedale San Giovanni
6500 Bellinzona, Switzerland
Phone 0041 91 811 9040
Fax 0041 91 811 9182
e-mail ielsg@eoc.ch
www.ielsg.org

Version n. 4.0 01 February 2017

PROTOCOL SIGN-OFF PAGE

The protocol IELSG 42 was accepted by the IELSG Board of Directors on February 23th 2013 and has passed the recommended review process for IELSG trials.
The final protocol amended, version 4.0, is dated February 01, 2017, 2016

IELSG President:

Name: Prof. Franco Cavalli
Date: February 01, 2017 Signature:

STUDY ACKNOWLEDGEMENT

A signed copy of this page must be sent to the IELSG Central Office before patient enrollment.

Protocol IELSG 42
As investigator for this study, I understand that this protocol contains information that is confidential and proprietary to IELSG. I have received and read the above mentioned protocol and agree that it contains all necessary details for carrying out the study as described; I will conduct this protocol as outlined therein.
I will provide copies of this protocol and access to all information furnished by IELSG to study personnel under my supervision. I will discuss this material with them to ensure that they are fully informed about the investigational product and the study. I agree to keep accurate records on all patients information (CRFs and Patients' informed consent statement) and all other information collected during the study for a minimum period of 10 years.
I agree not to publish all or any part of the results of the study carried out under this protocol, without the prior written consent of IELSG.
All parties agree to ensure direct access to examine, analyze, verify and reproduce source data / documents, and reports from all trial related sites for the purpose of monitoring and auditing, and inspection by domestic and foreign regulatory authorities.

……………………………………………………………….. ………………………………………………….. ……………………………..
Investigator (printed name) Signature Date

Institution, Address, Phone Number* ………

………..

………..

IELSG Representative Prof. Dr. med. Franco Cavalli Date: February 01, 2017

* If the address or phone number of the investigator changes during the course of the study, written notification will be provided by the investigator to the sponsor and will not require protocol amendment(s).

LIST OF ABBREVIATIONS / DEFINITION OF TERMS

AE	Adverse event
ARA-C	Cytarabine
ASCT	Autologous stem cell transplant
AUC	Area under the curve
BBB	Blood-brain barrier
BCNU	Carmustine
BS	Body surface
BW	Body weight
CMV	Cytomegalovirus
CNS	Central nervous system
CR	Complete remission
CRF	Case report form
CSF	Cerebrospinal fluid
CT	Computerized tomography
CTC	Common toxicity criteria
DFS	Disease-free survival
DLBCL	Diffuse large B-cell lymphoma
ECOG	Eastern Cooperative Oncology Group
HBV	Hepatitis B virus
HCV	Hepatitis C virus
HDC	High Dose Chemotherapy
HIV	Human immunodeficiency virus
ICH-GCP	Guideline for good clinical practice of the "International Conference on Harmonization"
IIT	Investigator initiated trial
IPI	International Prognostic index
LDH	Lactic dehydrogenase
LVEF	Left ventricular ejection fraction
mm^3	cubic millimeter
MRI	Magnetic resonance imaging
MTX	Methotrexate
NHL	Non-Hodgkin lymphoma
NOS	Not otherwise specified
NRM	Non-relapse mortality
OS	Overall survival
PCNSL	Primary CNS Lymphoma
PET	Positron emission tomography
PFS	Progression-free survival
PMN	Polymorphnuclear neutrophilic leukocytes
PR	Partial response
R-CHOP	Rituximab, Cyclophosphamide, Doxorubicin, Vincristine, Prednisolone
SADR	Serious adverse drug reaction
SAE	Serious adverse event
SDV	Source data verification
SOP	Standard operating procedure
VP16	Etoposide
WBRT	Whole-brain radiation therapy

TABLE OF CONTENTS

1 INTRODUCTION, BACKGROUND AND STUDY RATIONALE I
1.1 Introduction I
1.2 Background I
1.3 Study Rationale II
2 OBJECTIVES OF THE TRIAL II
2.1 Objectives II
2.2 Endpoints II
2.2.1 Primary Endpoint (for more details see item #11.3) II
2.2.2 Secondary Endpoints II
3 TRIAL DESIGN III
4 TRIAL DURATION AND TERMINATION TIMELINES IV
5 SELECTION OF PATIENTS IV
5.1 Inclusion criteria IV
5.2 Exclusion criteria V
6 REGISTRATION PROCEDURES V
7 STUDY DRUGS V
7.1 Drug Supply V
7.1.1 Methotrexate (MTX) V
7.1.2 Cytarabine (Ara-C) VI
7.1.3 Rituximab VI
7.1.4 Ifosfomide, carboplatin and etoposide (VP16) VII
7.1.5 Thiotepa VII
7.1.6 Carmustine (BCNU) VIII
7.2 Expected toxicity VIII
7.2.1 Expected adverse events with methotrexate VIII
7.2.2 Expected adverse events with cytarabine VIII
7.2.3 Expected adverse events with rituximab VIII
7.2.4 Expected adverse events with carmustine (BCNU) VIII
7.2.5 Expected adverse events with thiotepa IX
7.2.6 Expected adverse events with ifosfamide IX
7.2.7 Expected adverse events with etoposide (VP16) IX
7.2.8 Expected adverse events with carboplatin IX
7.3 Dose Modification IX
7.3.1 Chemotherapy dose modifications according to hematologic toxicity IX
7.3.2 Dose modifications of primary chemotherapy according to non-hematologic toxicity X
8 TREATMENT PLAN X
8.1 Treatment plan and duration X
8.1.1 Primary Chemoimmunotherapy X
8.1.1.1 Leukapheresis and cryopreservation XI
8.1.2 Conditioning and ASCT XII
8.1.3 Treatment of residual disease (post-ASCT) XII
8.1.4 Treatment of primary progressive disease XII
8.2 Criteria for withdrawal from study XII
8.3 Precaution, concomitant medications and not permitted treatments XIII
8.3.1 Prior and concomitant medication - antimicrobial prophylaxis XIII
8.3.2 Evaluation of infectious agents, antimicrobial and antiviral prophylaxis and high-dose Ig treatment XIII
8.3.3 Transfusional therapy XIII

8.3.4 Live Vaccines ... XIV
9 SAFETY ... XIV
9.1 Definition of Adverse Event ... XIV
9.1.1 Drug – Adverse Event Relationship ... XIV
9.2 Definition of Serious Adverse Event ... XIV
9.2.1 Pregnancy and Exposure in Utero ... XV
9.2.2 Exposure During Lactation ... XVI
9.2.3 Serious Adverse Drug Reactions (SADRs) ... XVI
9.2.4 Suspected Unexpected Serious Adverse Reactions (SUSARs) ... XVI
9.3 Procedures for AE, SAE and SUSAR Reporting ... XVI
10 CLINICAL EVALUATION, LABORATORY TESTS AND FOLLOW-UP ... XVII
10.1 Pretreatment ... XVII
10.2 During treatment ... XVII
10.3 After the end of the treatment ... XVII
10.4 After the progression of the disease ... XVII
11 EFFICACY ASSESSMENT ... XX
11.1 Criteria of evaluation ... XX
11.1.1 Schedule and methods of disease evaluation ... XX
11.1.2 Definition of response ... XX
11.1.3 Duration of the response ... XX
11.1.4 Patient without any response assessment ... XX
11.2 Transplantation Efficacy measures ... XXI
11.2.1 Characterization of the stem cell graft ... XXI
11.2.2 Definition of engraftment ... XXI
11.2.3 Evaluation of engraftment ... XXI
11.2.4 Documentation of transfusions (platelet and erythrocyte concentrates) ... XXI
11.2.5 Screening ... XXI
11.2.6 Initial examinations ... XXI
11.2.7 Interim examinations ... XXII
11.2.8 Appropriateness of measures ... XXII
11.2.9 Primary Engraftment efficacy ... XXII
11.3 Definition of Endpoints ... XXII
11.3.1 Primary endpoint ... XXII
11.3.2 Secondary endpoints ... XXII
12 STATISTICAL METHODS ... XXIII
12.1 Statistical design and sample size ... XXIII
12.2 Preliminary safety analysis ... XXIII
13 PATHOLOGY ... XXIII
13.1 Local pathology ... XXIII
13.2 Pathology review ... XXIII
14 ETHICAL CONSIDERATIONS ... XXIV
15 ADMINISTRATIVE CONSIDERATIONS ... XXIV
15.1 Monitoring and auditing ... XXIV
15.2 Data protection and archiving ... XXIV
15.3 Quality control and quality assurance ... XXV
16 PUBLICATION ... XXV
17 REFERENCES ... XXVI

1 INTRODUCTION, BACKGROUND AND STUDY RATIONALE

1.1 Introduction

Diffuse large B-cell lymphoma (DLBCL) rarely involves the central nervous system (CNS) at diagnosis or relapse, especially in patients with risk factors like increased serum LDH level, involvement of several extranodal sites, advanced stage, high IPI, and/or with involvement of particularly high-risk extranodal organs (i.e., testes, orbit, para-nasal sinuses) (1,2). In these lymphomas, CNS relapse usually occurs within the first year after diagnosis. CNS involvement at diagnosis or relapse is usually fatal despite effective therapy and CNS prophylaxis.
The great majority of patients with aggressive lymphomas and CNS relapse will eventually experience an associated systemic dissemination, concomitant or shortly later. As a consequence, the treatment should be effective against both the systemic and the CNS localization of the disease. A treatment against these targets should consider using drugs active in different cell-cycle phases and with a high blood-brain barrier (BBB) penetration ability; these drugs should also be administered with an active dose intensity/density schedule.
This is a prospective phase II trial aimed to assess the activity and feasibility of a new sequential chemoimmunotherapy program in patients younger than 71 years with DLBCL and CNS involvement either at presentation or relapse.

1.2 Background

NHL relapsing with CNS involvement after conventional chemotherapy has a poor prognosis. High-dose methotrexate (HD-MTX) is the most active cytostatic drug against NHL involving the CNS. At doses ≥3 g/m^2, MTX yields tumoricidal levels in both the brain parenchyma and the cerebrospinal fluid. Accordingly, in the recently concluded SCNSL1 trial, we assessed the feasibility and efficacy of a new HD-MTX-based chemotherapy combination, in which drugs with well-documented anti-lymphoma activity have been administered at high doses to increase BBB penetration and CNS bio-availability as well as to reduce potential cross-resistance with other drugs. This induction regimen followed by R-HDS (sequential high doses of cyclophosphamide, cytarabine and VP16) supported by BCNU-thiotepa/ASCT proved to be feasible in most patients ≤70 years old. Overall results were good, with 50% of the patients achieving a CR before transplantation and a significantly improved PFS. However, the unpredictable CNS bio-availability of cyclophosphamide resulted in some cases of early relapse; this took us to develop a new HD-MTX-based chemotherapy for induction, followed by R-ICE (rituximab, ifosfamide, carboplatin, etoposide) combination, a largely used salvage regimen in relapsed/refractory DLBCL, including drugs capable to cross BBB and not cross-resistant with antimetabolites; this new combination is named MARIETTA regimen. The main goals of adding R-ICE combination is to provide adequate treatment of CNS disease and to improve systemic disease control, which is a relevant issue considering that half of DLBCL patients with CNS involvement die of systemic progressive disease. The use of drugs with good CNS availability like ifosfamide, carboplatin and etoposide (i.e. ICE regimen) may result in improved efficacy, without a relevant increase in toxicity. In fact, recent studies showed that different ICE ± R regimens are associated with high response rates in patients with new diagnosed or relapsed/refractory PCNSL (3). With respect to SCNSL1 trial, the MARIETTA regimen includes the last version thiotepa-BCNU conditioning regimen, with higher doses of thiotepa, which has been largely tested in German centres with the same tolerability and higher efficacy (4). Similarly to the SCNSL1, the MARIETTA regimen offers the possibility to start with R-CHOP regimen in the case of extensive and life-threatening extra-CNS disease and to deliver whole-brain irradiation (WBRT) in patients with residual CNS disease after ASCT.

1.3 Study Rationale

This new sequential chemoimmunotherapy program called "MARIETTA" is based on two basic anti-lymphoma chemotherapy principles: the positive effect of dose intensity/density and of combination of cytostatics active in different cell-cycle phases. MARIETTA regimen is also based on the positive experience on primary CNS lymphoma with high doses of antimetabolites (5), the encouraging preliminary results with high-dose ifosfamide (3), the well-documented efficacy of R-ICE regimen as induction treatment for patients with relapsed/refractory DLBCL, and positive effect of consolidation with BCNU-thiotepa-based conditioning supported by ASCT in patients with primary CNS lymphoma (4). MARIETTA drugs have been selected both on their well-documented anti-lymphoma activity as well as their increased BBB penetration and CNS bioavailability. Importantly, sequential administration of R-ICE may result in reduced cross-resistance with antimetabolites, with a consequent improved efficacy.

2 OBJECTIVES OF THE TRIAL

2.1 Objectives

This is an open, non comparative, multicentre phase II trial, to evaluate the efficacy and feasibility of a new sequential combination of HD-MTX-AraC-based chemoimmunotherapy, followed by R-ICE regimen, and by high-dose chemotherapy supported by ASCT.

2.2 Endpoints

2.2.1 Primary Endpoint (for more details see item #11.3)

1-year progression-free survival (PFS)

2.2.2 Secondary Endpoints

a. Complete remission rate before autologous stem cell transplantation
b. Response duration
c. Overall survival
d. Safety

3 TRIAL DESIGN

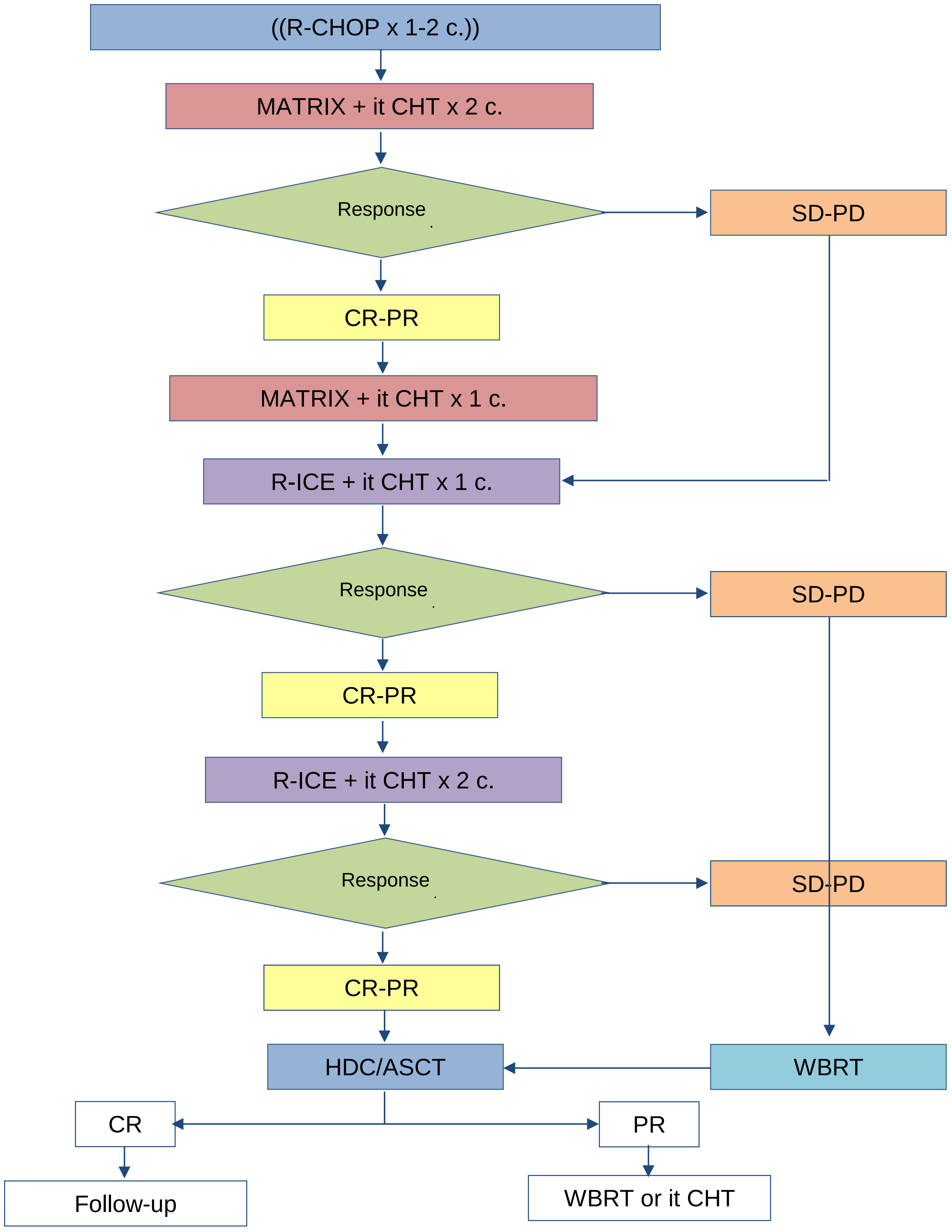

4 TRIAL DURATION AND TERMINATION TIMELINES

The inclusion of patients is planned to start in Q3 2014 and will stop after the inclusion of 76 assessable patients, which is expected in Q3 2017. End of trial treatment (last patient, last visit) is expected for Q3 2018.
All patients will be followed up for 5 years. The trial team, together with the head trial coordination and the director may decide to stop the follow up period earlier. The trial will be terminated when the last patient has died or the follow up period was stopped early. Criteria for shortening the follow up period will be determined by the trial oversight groups at IELSG.

Accrual may be interrupted and/or the trial may be stopped early by the board based on the safety/efficacy evaluations, as a result of an interim analysis (see section #12.2) or if new data become available which change assessment of risk/benefit.

5 SELECTION OF PATIENTS

5.1 Inclusion criteria

14. Histologically confirmed diagnosis of diffuse large B-cell lymphoma
15. CNS involvement (brain, meninges, cranial nerves, eyes and/or spinal cord) at diagnosis (concomitant to extra-CNS disease) or relapse after conventional chemo(-immuno)therapy
16. Diagnosis of CNS involvement either by brain biopsy or CSF cytology examination. Neuroimaging alone is acceptable when stereotactic biopsy is formally contraindicated or when the disease has been previously histologically documented in other areas and the CNS localization is concomitant with a diffuse progression of systemic disease.
17. No previous treatment with high-dose methotrexate-based chemotherapy and/or brain irradiation.
18. Age 18-70 years
19. ECOG performance status 0-3
20. Adequate bone marrow (Platelets count ≥100x10^3/mm^3, hemoglobin ≥9 g/dL, neutrophils count ≥1.5x10^3/mm^3), renal (creatinine clearance ≥60 mL/min), cardiac (LVEF ≥50%), and hepatic function (total serum bilirubine ≤3 mg/dL, AST and ALT and GGT ≤2.5 per upper normal limit value), unless the abnormality is due to lymphoma infiltration
21. Absence of HIV infection and of detectable HCV-RNA and/or HBsAg and/or HBV-DNA
22. No concurrent malignancies. Previous malignancies are accepted if surgically cured or if there was no evidence of disease in the last 3 years at a regular follow-up
23. Absence of any familial, sociological or geographical condition potentially hampering compliance with the study protocol and follow-up schedule
24. Female patients must be non-pregnant and non-lactating. Sexually active patients of childbearing potential must implement adequate contraceptive measures during study participation. Highly effective contraceptive precautions are required until 12 months after the completion of trial treatment. Pregnancy test must be performed within 7 days of commencing study treatment.
25. No treatment with other experimental drugs within the 6 weeks previous to enrolment
26. Given written informed consent prior to any study specific procedures, with the understanding that the patient has the right to withdraw from the study at any time, without any prejudice. Informed consent signed by a patient's guardian is acceptable if the patient is not able to decide inclusion in the study due to cognitive impairment

5.2 Exclusion criteria

8. Other lymphoma categories other than diffuse large B-cell lymphoma. In particular, patients with primary mediastinal lymphoma, intravascular large B-cell lymphoma or leg-type large B-cell lymphoma are excluded.
9. Patients with positive flow cytometry examination of the CSF, but negative results in CSF conventional cytology, and without any other evidence of CNS disease.
10. Patients with exclusive CNS disease at presentation (primary CNS lymphoma) are excluded
11. Previous treatment with support of autologous or allogeneic stem cells/bone marrow transplantation.
12. Symptomatic coronary artery disease, cardiac arrhythmias not well controlled with medication or myocardial infarction within the last 6 months (New York Heart Association Class III or IV heart disease)
13. Any other serious medical condition which could impair the ability of the patient to participate in the trial.
14. Any contraindications as identified in relation to the relevant SmPCs

6 REGISTRATION PROCEDURES

Patients fulfilling the eligibility criteria and for whom informed consent signed by the patient or his/her guardian is obtained will be centrally registered at the following website:

https://openclinica.eoc.ch/OpenClinica

The login data will be provided by IELSG.
Treatment should start within 7 days from registration.
A sequential identification number will be automatically attributed to each participating institution and to each patient registered in the trial. These numbers will identify the patient and must be included on all case report forms. In order to avoid identification errors date of birth will also be reported on the case report forms.

7 STUDY DRUGS

7.1 Drug Supply

Methotrexate, cytarabine, rituximab, Ifosfomide, carboplatin and etoposide (VP16), thiotepa, and Carmustine (BCNU) are considered standard treatment; they are commercially available worldwide and will be provided by each participating institute.

7.1.1 Methotrexate (MTX)

MTX functions as an antimetabolite by reversibly inhibiting dihydrofolate reductase, the enzyme that reduces folic acid to tetrahydrofolic acid.
The most favourable administration schedule for MTX in PCNSL remains to be defined, due to the wide range of different doses (1-8.4 g/m^2) used in prospective trials and to its frequent association with different drugs and/or RT. The timing of MTX administration has been analysed in a single small series and no significant difference in terms of survival or toxicity was observed between the administration of 3.5 g/m^2 every 3 weeks or every 10 days.
MTX enters the cells in part by an active transport mechanism and is bound as polyglutamate conjugates. During longer periods of drug exposure, a higher polyglutamate formation rate is observed and more cells enter into phase S, resulting in an increased cytotoxicity. In a study comparing 3- vs. 6-hour infusions of MTX, the former

was significantly associated with a higher response rate and increased CSF levels. The optimal MTX schedule seems to be an initial rapid administration to overcome the distribution phase of clearance followed by a 3-hour infusion for doses 5 g/ m^2.
Patients with renal or hepatic insufficiency require a reduced MTX dose. Peak serum levels occur within 30 to 60 min with parenteral doses; maximal myelosuppression occurs within 7 to 10 days; duration of tumor response and hematopoietic effects is 7 to 14 days; protein binding is 50%; cerebrospinal fluid concentrations are 1% of the simultaneous serum concentration; volume of distribution is 0.4 to 0.9 L/kg; elimination half-life is 8 to 15 h; minimal hepatic metabolism is followed with 48% to 100% excreted unchanged in the urine and 9% in the feces. Adverse effects include leukopenia, thrombocytopenia, anaemia, pancytopenia, vasculitis, neurotoxicity, headache, fever, paraplegia, cerebellar dysfunction, cranial nerve palsies, seizures, dementia, ataxia, drowsiness, paresis, colitis, toxic megacolon, gingivitis, anorexia, nausea, vomiting, diarrhea, hematemesis, melena, gastrointestinal bleeding, stomatitis, pseudomembranous colitis, nephrotoxicity, cystitis, menstrual dysfunction, oligospermia, infertility, hepatotoxicity, conjunctivitis, blurred vision, interstitial pneumonitis, alopecia, rash, toxic epidermal necrolysis, phototoxicity, systemic lupus erythematosus, and hypersensitivity with anaphylaxis. In addition, MTX has been associated with tumor lysis syndrome and potentially life-threatening or fatal opportunistic infections.
MTX is an effective antineoplastic agent against a variety of cancers, such as breast cancer, acute lymphoblastic leukemia, osteogenic sarcoma, head and neck cancer, ovarian cancer, non-Hodgkin's lymphoma, colorectal carcinoma, and Hodgkin's lymphoma. In addition, the drug is used extensively for severe cases of recalcitrant psoriasis, severe rheumatoid arthritis, systemic lupus erythematosus, and inflammatory bowel disease; however, due to potential toxicity, it should be used with caution in these patients.

7.1.2 Cytarabine (Ara-C)

Ara-C is a synthetic antimetabolite that is cell-cycle specific. Ara-C is cytotoxic primarily to cells in the S-phase. High-dose therapy consists of 1-3 gr/m^2 every 12 hours. Following IV administration, Ara-C is widely distributed to areas including the CNS and tears. Ara-C is metabolized in the liver to an inactive metabolite; both Ara-C and its metabolite are excreted in the urine. The elimination half-life is between 1 and 3 hours. The major toxic effect of Ara-C is myelosuppression resulting in megaloblastic changes in erythropoiesis and reticulocytopenia. Other adverse effects include neuropathies, GI distress, hepatic toxicity, and hypersensitivity. Ara-C is useful in various neoplastic disorders including chronic myelocytic leukemia, lymphoblastic leukemia, acute lymphocytic leukemia, acute non-lymphocytic leukemia, meningeal leukemia, and non-Hodgkin’s lymphomas. Other disease states in which Ara-C has been used include herpes virus infections and psoriasis.
Ara-C has been used in different combinations with HD-MTX in the treatment of PCNSL, mostly with encouraging results. The clinical benefit of the addition of HD-AraC to HD-MTX has been suggested by a large retrospective series and a meta-analysis of 19 prospective trials and has been confirmed by the first worldwide randomized trial with complete accrual in PCNSL (5-7). As main conclusion from that trial, the combination of HD-MTX and HD-araC could be used as control arm for future randomized trials assessing new chemotherapy combinations for newly diagnosed PCNSL.
Importantly, cytarabine free and intraliposomal cytarabine (Depocyte®) can be delivered by intrathecal route to treat meninges and/or to prevent lymphomatous dissemination in those organs. Doses and schedules are reported in section #8.1.

7.1.3 Rituximab

Rituximab is a chimeric monoclonal antibody directed against the B-lymphocyte antigen CD20. This antibody has been largely used in the treatment of B-cell lymphomas with excellent results. In particular, the addition of rituximab to CHOP chemotherapy regimen has been associated with a significantly improvement in outcome in patients with DLBCL (8). This is a relevant aspect considering that this lymphoma category constitutes the most

common histological form of PCNSL. Nevertheless, rituximab has been only anecdotally used in PCNSL patients considering that there are many doubts about its capability to cross the BBB (9). Feasibility of a combination of HD-MTX and rituximab has been demonstrated in a prospective series (10), but its real contribution in the management of PCNSL remains to be defined. Rituximab delivery can be complicated by cerebral oedema; this antibody should not be given any more if followed by signs of cerebral oedema. In the present trial, patient with signs of cerebral oedema should not be taken off study for this reason only, but the study treatment should be continued without rituximab.
Rituximab is usually a well tolerated agent, with some forms of infusion-related reactivity (rash, broncospasm, allergic processes, fever, hypotension). Severe events are rare and could be related to a high tumor burden, and, with the single exception of intravascular large B-cell lymphoma, they have not been reported in lymphoma patients with CNS involvement.

7.1.4 Ifosfomide, carboplatin and etoposide (VP16)

Ifosfamide, carboplatin and etoposide are active drugs largely used in aggressive extra-CNS lymphomas and have the capability to cross the normal BBB reaching cytotoxic levels in the CSF and brain parenchyma (11,12). A combination of etoposide, ifosfamide and cytarabine (VIA) was evaluated in 16 immunocompetent patients with refractory/recurrent PCNSL (13). The CR rate was 37% and 1-y OS was 41%. A series of six patients with recurrent or refractory PCNSL treated with dexametasone, etoposide, ifosfamide and carboplatin (DeVIC regimen) showed a total response rate of 83% (14). More recently, a combination named R-IE (rituximab 375 mg/m^2 day 0; ifosfamide 2 g/m^2/d days 1-3; etoposide 250 mg/m^2 day 1) was used in 22 patients with relapsing or refractory PCNSL treated ten Italian Centers. Toxicity was manageable, with a single case of grade-4 non-hematologic toxicity, with a response rate of 41%. R-IE has made possible stem cell collection and consolidation with HDC/ASCT in one-third of patients. DeVIC chemotherapy combination has been also retrospectively evaluated as first-line treatment in 21 PCNSL patients, followed by WBRT consolidation. The ORR after the first two cycles of chemotherapy was 95.2%, the median PFS in all patients was 37.4 months and the median duration of OS was 47.8 months (15), thus confirming activity and efficacy of these drugs in PCNSL.

7.1.5 Thiotepa

Thiotepa is a cytotoxic agent of the polyfunctional alkylating type (more than one reactive ethylenamine group), related chemically and pharmacologically to nitrogen mustard. Its radiomimetic action is believed to occur through the release of ethylenamine radicals which, like irradiation, disrupt the bonds of DNA. One of the principal bond disruptions is initiated by alkylation of guanine at the N-7 position, which severs the linkage between the purine base and the sugar and liberates alkylated guanines. Thiotepa has been tried with varying results in the palliation of a wide variety of neoplastic diseases. However, the most consistent results have been seen in the following tumours: adenocarcinoma of the breast and ovary; for controlling intracavitary effusions secondary to neoplastic diseases of various sierosal cavities; for the treatment of superficial papillary carcinoma of the urinary bladder. Some efficacy has been proved for Hodgkin's disease and other lymphomas.
Thiotepa is highly toxic to the hematopoietic system, and bone-marrow depression has to be expected. Weekly blood and platelet counts are recommended during therapy and for at least 3 weeks after therapy discontinuation. Other adverse effects include: fatigue, weakness, allergic reactions, nausea, vomiting, abdominal pain, anorexia, dysuria, urinary retention, dizziness, headache, blurred vision, dermatitis, alopecia, amenorrhea, interference with spermatogenesis.
Thiotepa is capable of cross-linking the DNA within a cell and changing its nature. The replication of the cell is, therefore, altered, and thiotepa may be described as mutagenic. Effective contraception should be used during thiotepa therapy if either the patient or the partner is of childbearing potential. In patients treated with thiotepa, cases of myelodysplastic syndromes and acute nonlymphocytic leukemia have been reported. There is no known antidote for overdosage with thiotepa.

A single-arm phase II trial assessing the chemotherapy combination named “MATILDE” included thiotepa (16). This combination has been associated with an ORR of 72% and a CRR of 46%, with a 5-yr OS of 42% and a persistent plateau in the survival curve. Thiotepa has been used in combination with other alkylating agents as conditioning regimens for ASCT in patients with PCNSL, both at diagnosis or relapse (4).

7.1.6 Carmustine (BCNU)

Carmustine is an alkylating agent that belongs to the Nitrosoureas group. These agents act by the process of alkylation to inhibit DNA repair. The nitrosoureas can cross the blood-brain barrier and are therefore used to treat brain tumors. BCNU is also effective as a single agent against lymphomas and Hodgkin’s disease.
Bone marrow suppression, notably thrombocytopenia and leukopenia, is the most common and severe toxic effect of BCNU. Myelosuppression appears later (14-28 days) and last longer (up to 6-8 weeks) than most other cytotoxic drugs. Other less frequent adverse side-effects are nausea, vomiting, diarrhea, pulmonary fibrosis, skin flashing, amenorrhea and interference with spermatogenesis. Secondary carcinogenesis has occasionally been reported, as well as secondary acute myeloid leukemias.
This drug has been largely used in primary brain tumours, and, importantly, in PCNSL, both at conventional doses and as part of conditioning regimens before ASCT (4).

7.2 Expected toxicity

Treatment side effects will be assessed separately for each course of chemotherapy. Acute side effects will be graded according to the “Common Toxicity Criteria” defined by the NCI (US) extended by the NCIC (Canada) version 4.03. Safety and toxicity data will be collected on electronic CRF and centrally collected and monitored at the IELSG secretariat as for all the other trial parameters.

7.2.1 Expected adverse events with methotrexate

The most frequently reported adverse reactions of high dose methotrexate include ulcerative stomatitis, myelosuppression, nausea and vomiting. Acute encephalopathy and toxic nephropathy have been reported, too.

7.2.2 Expected adverse events with cytarabine

The most frequently reported adverse reactions of cytarabine include myelosuppression, febrile neutropenia, thrombocytopenia, bleeding, nausea and vomiting, stomatitis. Cerebellar toxicity has been reported in association with high dose therapy. Conjunctivitis, skin reactions (rash) and liver toxicity can occur too.

7.2.3 Expected adverse events with rituximab

The most serious adverse reaction with rituximab include: infusion reactions, mucocutaneous reactions, hypersensitivity reactions, cardiac arrhythmias, angina, and renal failure. Mild to moderate infusion reactions consisting of fever and chill/rigors occurred in the majority of patients during the first infusion. Other frequent infusion reaction symptoms included nausea, pruritus, angioedema, asthenia, hypotension, headache, bronchospasm, throat irritation, rhinitis, urticarial, rash, vomiting, myalgia, dizziness and hypertension and reactivation of HBV infection.

7.2.4 Expected adverse events with carmustine (BCNU)

The most frequently reported adverse reaction of BCNU include myelosuppression (appears later and lasts longer than most other cytotoxic drugs), thrombocytopenia, febrile neutropenia, nausea, vomiting, diarrhoea, stomatitis, mucositis, neurological symptoms. Lung fibrosis appears in about 1/3 of patients treated with very high dose of BCNU. Amenorrhea is frequent and interference with spermatogenesis is more than occasional.

7.2.5 Expected adverse events with thiotepa

The most frequently reported adverse reactions of thiotepa include myelosuppression, thrombocytopenia, febrile neutropenia, nausea and vomiting. Amenorrhea and interference with spermatogenesis are expected.

7.2.6 Expected adverse events with ifosfamide

The most frequently reported adverse reactions of ifosfamide include myelosuppression, hemorrhagic cystitis, stomatitis, nausea and vomiting. Acute encephalopathy and toxic nephropathy have been reported, too. Amenorrhea is frequent and interference with spermatogenesis is more than occasional.

7.2.7 Expected adverse events with etoposide (VP16)

The most frequently reported adverse reactions of etoposide include myelosuppression, febrile neutropenia, thrombocytopenia, bleeding. Neurotoxicity, mucositis and vomiting are uncommon.

7.2.8 Expected adverse events with carboplatin

The most serious adverse reaction with carboplatin include myelosuppression, thrombocytopenia, febrile neutropenia, renal failure, ototoxicity, vomiting.

7.3 Dose Modification

7.3.1 Chemotherapy dose modifications according to hematologic toxicity

In case of inadequate bone marrow recovery, that is ANC <1.0x10^3/mm^3 and platelets <90x10^3/mm^3 , on the intended day of re-treatment, the start of the next cycle could be delayed for a maximum of 4 weeks. Thereafter, chemotherapy has to be discontinued, and patient referred to alternative salvage treatment according to physician's preferences.

The dose of cytostatic drugs will be determined according to the nadir neutrophil or platelet counts of the previous course as follows:

Nadir neutrophils (x10^3/mm^3)	MATRIX	R-ICE	Nadir platelets (x10^3/mm^3)	MATRIX	R-ICE
≧ 2	Unchanged	Unchanged	≧ 125	Unchanged	Unchanged

1.5-1.999	Unchanged	Unchanged	75 -124.999	Unchanged	Unchanged
1.0-1.499	Unchanged	Unchanged	50-74.999	Unchanged	Unchanged
0.5-0.9	Unchanged	Unchanged	25-49.999	Unchanged	Unchanged
<0.5	25% decrease of Ara-C if complicated with infection*	25% decrease of ifosfamide if complicated with infection	< 25	25% decrease of Ara-C if complicated with bleeding*	25% decrease of ifosfamide if complicated with bleeding

*Ara-C 25% dose reduction consists on the omission of the 4th cumulative dose (2nd dose of day 3).

7.3.2 Dose modifications of primary chemotherapy according to non-hematologic toxicity

For CTCAE grade 2 or less non-hematological toxicity, no dose reductions will be required. For CTC (version 4.03) grade 3-4 non-hematological toxicity, the total dose of drugs to be administered for the next course will be reduced as follows:

Toxicity	Grade 3		Grade 4	
	MATRIX	R-ICE	MATRIX	R-ICE
Cardiovascular	Interruption	Interruption	Interruption	Interruption
Coagulation	Unchanged	Unchanged	25% decrease for Ara-C	25% decrease for ifosfamide
Gastrointestinal	Unchanged	Unchanged	25% decrease for MTX and Ara-C	25% decrease for all cytostatics
Renal	25% decrease for MTX	25% decrease for all cytostatics	25% decrease for MTX and Ara-C	25% decrease for all cytostatics
Hepatic	25% decrease for all cytostatics	25% decrease for all cytostatics	25% decrease for all cytostatics	25% decrease for all cytostatics
Pulmonary	Unchanged	Unchanged	25% decrease for MTX and Ara-C	25% decrease for all cytostatics

Rituximab infusion reactions will be managed according to institutional guidelines.

Neurotoxicity (chemical or aseptic meningitis/arachnoiditis) will be monitored and intrathecal drug delivery will be reduced or interrupted according to grade and duration on the base of physician's preference. In the case of signs and symptoms of chemical arachnoiditis, steroid therapy delivered before and after intrathecal chemotherapy will be potentiated (double daily dose for 5 days).

8 TREATMENT PLAN

8.1 Treatment plan and duration

Experimental treatment will be started within three weeks from baseline assessment of target lesions. Treatment includes 6 courses of chemoimmunotherapy (see Figure), the first three courses with an HD-MTX-based combination followed by other three courses of R-ICE combination and finally a BCNU-thiotepa-containing conditioning and subsequent ASCT. The interval between the first day of two consecutive courses will be three weeks. The interval between the last chemoimmunotherapy course and conditioning will be 4-6 weeks.

8.1.1 Primary Chemoimmunotherapy

MATRIX (courses 1, 2, 3)*
***Patients with concomitant, extensive and life-threatening extra-CNS disease can start the chemoimmunotherapy program with 1 or 2 courses of conventional R-CHOP regimen (to be given as per local practice) after the baseline assessments have been completed.**

day 0:	Rituximab 375 mg/m^2 as conventional IV infusion
day 1:	Methotrexate 3.5 g/m^2, the first 0.5 g/m2 in 15 min. + 3 g/m^2 in 3-hr IV infusion
days 2 and 3:	Cytarabine 2 g/m^2 every 12 hours, in 1-hr IV infusion
	Folinic rescue (levo-folinic) 15 mg/m^2 IV every 6 hrs. for 12 doses (adjusted on MTX level)** (details see below)
day 4:	Thiotepa 30 mg/m^2 in 30 minutes IV infusion
day 5:	Intrathecal liposomal cytarabine 50 mg*** (details see below)
days 6 to 12:	G-CSF (both filgastrim or lenogastrim can be used, as per local practice)

***LEUCOVORIN RESCUE AND POST-MTX HYDRATION*
Folinic (THF) rescue must commence 24 hours after the start of MTX infusion. Levo-folinic acid will be administered at a dose of 15 mg/m2 (folinic 30 mg/m2) intravenous push/PO every six hours for 12 doses. The post MTX hydration (including the solutions used to administrate Ara-C, antiemetics and other drugs) should reach a total volume of 2000 ml (or as per local practice).

MTX SERUM LEVEL DETERMINATION
MTX serum level determinationis to be taken at 48 hours post methotrexate., and it will be repeated every 24 hours until a concentration < 5 x 10-8M/L is reached. In the case of high MTX serum levels persisting after 48 hours (or more) after the end of infusion, leucovorin rescue will be modified according to MTX levels as follows:
if MTX < 5 x 10-7 M/L levofolinic acid 15 mg/m2/6 hours (folinic acid – racemic - 30 mg/m2/6hours)
if MTX < 1 x 10-6 M/L levofolinic acid 50 mg/m2/6 hours (folinic acid – racemic - 100 mg/m2/6hours)
if MTX > 1 x 10-6 M/L levofolinic acid100 mg/m2/6 hours. (folinic acid – racemic- 200 mg/m2/6hours)
The rescue at 72 and 96 hours follows the dosing guidance for 48 hours.

****If liposomal cytarabine is not available, standard intrathecal chemotherapy with "methotrexate 10-12 mg + cytarabine 40-50 mg + hydrocortisone 50 mg" can be administered (doses to be consistent with local institutional guidelines). Oral steroids (as per local practice) are suggested for 2-5 days after intrathecal liposomal cytarabine delivery to prevent chemical or aseptic meningitis/arachnoiditis.*

R-ICE (courses 4, 5, 6)

day 1:	Rituximab 375 mg/m^2 as conventional IV infusion
days 1-2-3:	Etoposide 100 mg/m^2/d in 500-1000ml (as per local practice). over 30-60minutes IV infusion
day 2	Ifosfamide 5 g/m^2 in 1,000 mL in 24-hour IV infusion with MESNA support (as per local practice)
	Carboplatin AUC= 5 (capped dose of carboplatin is 800 mg, total dose)) in 500 mL in 1-hour IV infusion
day 4:	Intrathecal liposomal cytarabine 50 mg*

**If liposomal cytarabine is not available, standard intrathecal chemotherapy with "methotrexate 10-12 mg + cytarabine 40-50 mg + hydrocortisone 50 mg" can be administered (doses to be consistent with local institutional guidelines). Oral steroids are suggested for 2-5 days after intrathecal liposomal cytarabine delivery to prevent chemical or aseptic meningitis/arachnoiditis.*

8.1.1.1 Leukapheresis and cryopreservation

Autologous peripheral blood stem cells (APBSC) will be collected after the 2nd course of MATRIX, starting day 10, absolute CD34+ cell count per L of blood are to be determined every day. The objective is to harvest a minimum of 5 x 10^6 CD34+ cells/kg of body weight with as few as possible leukapheresis sessions during consecutive days. CD34+ cells will be collected, processed and stored according to conventional guidelines. Plerixafor use is allowed according to institutional guidelines.
In the case for clinical or organizational reasons APBSC harvest is not possible after the 2nd MATRIX course, leukapheresis could be performed after the 3rd MATRIX course or after the 1st R-ICE course. APBSC can be

collected after the first R-ICE course in selected patients with extensive or bulky extra-CNS disease or with bone marrow infiltration or with progressive disease during MATRIX.

8.1.2 Conditioning and ASCT

day -6: BCNU 400 mg/m^2 in 500 mL in 1-2hr infusion*,
day -5 and -4: Thiotepa 5 mg/kg in saline sol. in 2-hrs infusion every 12 hours
day 0: Auto-transplantation (ASCT): reinfusion of 5 x 10^6 CD34+ cells/kg
* In case of BCNU unavailability, the recommended conditioning regimen (Phase IV) is:
day -6 and -5: Thiotepa 5 mg/kg in saline sol. by 2-hrs infusion
day -4, -3 and -2: Busulfan 3.2 mg/kg (administered in four doses per day corresponding to 0.8 mg/kg each dose) by 2-hr infusion or 3.2mg/kg as a once daily infusion given over 3 hours)
Clonazepam prophylactically from the day before busulfan therapy to the day after completion of busulfan therapy. (*different prophylaxis schemes, according to institutional guidelines, are accepted)*
day 0: *Auto-transplantation* : reinfusion of 5 x 10^6 CD34+ cells/kg harvested during phases II-III

8.1.3 Treatment of residual disease (post-ASCT)

Therapeutic program will be completed with whole-brain irradiation (“WBRT” in the figure) 36 Gy + tumor-bed boost 10 Gy in patients with residual disease in the parenchymal brain/cerebellum. WBRT will start within one month since response assessment (MRI post ASCT)., Whole-brain will be irradiated by two opposite lateral fields including the first two cervical vertebras and the posterior two thirds of the eyes, which must be shielded after 30 Gy. Tumor bed (boost with 1 - 2 cm of margin other than initial enhanced lesion) will be irradiated by 2 to 4 isocentric treatment fields based on tumor location, with all portals treated per each RT session. Photons of 4 - 10 MeV, 180 - 200 cGy per day, 5 weekly fractions will be employed. Patients with positive CSF cytology examination after ASCT will be managed with intrathecal / intraventricular chemotherapy (“it CHT” in the figure) with methotrexate 12 mg + cytarabine 50 mg + hydrocortisone 50 mg days 1 and 8; and/or thiotepa 10 mg + rituximab 25 mg days 4 and 11 of every months, for three months.

8.1.4 Treatment of primary progressive disease

Patients who will experience progressive disease at any time during MATRIX chemotherapy will proceed with R-ICE chemotherapy, conditioning and ASCT (Figure). Patients who will experience progressive disease at the CNS during R-ICE will be referred to WBRT (according to institutional guidelines, suggested 40-45 Gy to the whole brain), followed by conditioning and ASCT in the case of response. Patients who will experience progressive disease at extra-CNS organs during R-ICE will be considered off study, and treated according to physician’s preferences.

8.2 Criteria for withdrawal from study

The patient may drop out of the clinical study at any time without stating reasons. This may not have any negative consequences for the patient’s further treatment.
Possible reasons for withdrawal of the patient are:

- Withdrawal of consent at any time
- Patient’s death

In the case of patient’s choice to withdraw, it will be censored but not regarded as an event (i.e. not treatment failure). Patients for whom premature termination of the study before day +100 after ASCT can be foreseen or

who have been withdrawn from the study should undergo the 'day +100'- examination if possible. Patients who terminate prematurely between day +100 and 'End of follow-up' should undergo a “final examination”. Findings of these examinations have to be documented in the CRF.

Patient will be considered off study in case of confirmed relapse or severe treatment-related toxicity and, consequently, candidate to salvage therapy. Neurological deterioration without radiological assessment may not be considered progression of disease nor a sufficient reason to stop treatment.

All patients will be followed up to 5 years. No trial-specific assessments will be performed after progression.

8.3 Precaution, concomitant medications and not permitted treatments

8.3.1 Prior and concomitant medication - antimicrobial prophylaxis

The following drugs can be delivered: antiemetics, analgesics, antibiotics, anticonvulsants, sedatives, anti-hyperuricemic agents as well as other therapies to control metabolic and malnutrition disturbances. The type and doses of anticonvulsants and corticosteroids has to be accurately registered. Corticosteroids should be weaned and stopped during the 1st cycle of MATRIX, according to clinical requirements.
The use of other hepatotoxic (azathioprine, retinoids, sulfasalazin) and renal toxic (NSAIDs, cotrimoxazole, allopurinole, aciclovir) agents should be avoided during MTX exposure. Within two days prior/after MTX administration proton pump inhibitors should be stopped and contrast agents should not be administered.
Antimicrobial prophylaxis should follow Institutional guidelines since the variability in endemic or epidemic distribution of infectious agents. However, oral antiviral (Acyclovir 400 mg bid), antifungine (Fluconazole 200 mg/d) and antipneumocystic (Trimethoprim 160 mg and sulfamethoxazole 800 mg, thrice per week) prophylaxis are suggested. Conventional doses of G-CSF from day 6th to 12th of every course associated with antibiotic prophylaxis as per local practice (same period). Oral administration can be replaced by parenteral route according to clinical requirements. Interruption of oral antimicrobial prophylaxis from day -1 to day +5 of every chemoimmunotherapy course is suggested to avoid adverse pharmacological interactions. Administration of immunoglobulines (200 mg/mq; every 10 days, for 4 times) is suggested in case of hypogammaglobulinemia due to rituximab. Medication given between day -6 and day 21 must be recorded in the CRF with the period of administration.
Additional cytotoxic therapy, biological responsive modifiers and drugs possibly interfering in the action or pharmacokinetics of MTX, Ara-C, rituximab, ifosfamide, VP-16, carboplatin, BCNU or thiotepa must be avoided.

8.3.2 Evaluation of infectious agents, antimicrobial and antiviral prophylaxis and high-dose Ig treatment

IgG, IgA and IgM serum levels will be assessed regularly until six months after ASCT and a prophylactic high-dose Ig infusion (0.4 g/Kg) will be administered every 4 weeks if an IgG depletion was detected (values <0.5 g/dL). CMV-DNA PCR detection will be checked every week following ASCT until day +60 post transplant, and every two weeks until six months after transplant, as well. In case of fever of unknown origin or of pancytopenia after transplant it should be mandatory to check the CMV status. An eventual antiviral therapy will consists in Gancyclovir (5 mg/Kg bid) for at least 7 days or when two consecutive CMV-DNA controls are negative. In case of associated pancytopenia, Foscarnet has to be administered for at least 7 days or until two consecutive CMV-DNA controls are negative. Different dosage scheme of Foscarnet according to institutional guideline could be accepted. In case of no response after 10 days, Gancyclovir combined with Foscarnet has to be administered

8.3.3 Transfusional therapy

All transfusional products must be irradiated (30 Gy) to avoid any transfusional GVH disease.
Platelet transfusion will be performed when counts will be <10 x 109/L, or <20 x 109/L in case of fever or active bleeding. RBC transfusion will be performed with haemoglobin <8.0 g/dL or in selected patients with higher levels according to physician's preference.

8.3.4 Live Vaccines

The use of live vaccine, during experimental treatment and for 12 months during follow up period, is prohibited.

9 SAFETY

9.1 Definition of Adverse Event

Patients will be instructed by the investigator to report the occurrence of any adverse event.
An adverse event (AE) is any untoward medical occurrence in a patient administered a pharmaceutical product, which does not necessarily have a causal relationship with the treatment.
An adverse event can be any unfavourable and unintended sign (e.g., including an abnormal laboratory finding), symptom, or disease temporally associated with the use of the study drug, whether or not it is considered to be study drug related. This includes any newly occurring event or previous condition that has increased in severity or frequency since the administration of study drug.

9.1.1 Drug – Adverse Event Relationship

The causality relationship of study drug to the adverse event will be assessed by the Investigator as either Yes or No.
If there is a reasonable suspected causal relationship to the study medication, i.e. there are facts (evidence) or arguments to suggest a causal relationship, the drug-event relationship should be assessed as Yes and the AE is defined as Adverse Reaction (AR).
The following criteria should be considered in order to assess the relationship as Yes:

- Reasonable temporal association with drug administration
- It may or may not have been produced by the patient's clinical state, environmental or toxic factors, or other modes of therapy administered to the patient
- Known response pattern to suspected drug
- Disappears or decreases on cessation or reduction in dose
- Reappears on re-challenge

The following criteria should be considered in order to assess the relationship as No:

- It does not follow a reasonable temporal sequence from administration of the drug
- It may readily have been produced by the patient's clinical state, environmental or toxic factors, or other modes of therapy administered to the patient
- It does not follow a known pattern of response to the suspected drug
- It does not reappear or worsen when the drug is re-administered

9.2 Definition of Serious Adverse Event

A serious adverse event (SAE) is any adverse event, occurring at any dose and regardless of causality that:

- Results in death.
- Is life-threatening (Grade 4 according to CTCAE). Life-threatening means that the patient was at immediate risk of death from the reaction as it occurred, i.e., it does not include a reaction which hypothetically might have caused death had it occurred in a more severe form. (Exceptions: Grade 4 AEs not to be reported as SAEs: hematological toxicity and mucositis).
- Requires inpatient hospitalization or prolongation of existing hospitalization. Hospitalization admissions and/or surgical operations scheduled to occur during the study period, but planned prior to study entry are not considered AEs if the illness or disease existed before the patient was enrolled in the trial, provided that it did not deteriorate in an unexpected manner during the trial (e.g., surgery performed earlier than planned).
- Results in persistent or significant disability/incapacity. Disability is defined as a substantial disruption of a persons' ability to conduct normal life functions.
- Is a congenital anomaly/birth defect.
- Is an important medical event. An important medical event is an event that may not result in death, be life-threatening, or require hospitalization but may be considered a SAE when, based upon appropriate medical judgment, it may jeopardize the patient or subject and may require medical or surgical intervention to prevent one of the outcomes listed in the definitions for SAEs (e.g., any new malignancy other than a relapse of the current tumor need to be reported as SAE).

Lymphoma progression and death due to lymphoma progression are not considered as SAE.

9.2.1 Pregnancy and Exposure in Utero

If a patient becomes pregnant whilst on study treatment she must immediately cease treatment. In the case of pregnancy occurring during the course of the trial or within 90 days of discontinuing the study drug, the investigator must report the event to the IELSG operation office within the same timelines as a SAE and classify it as an 'other medically significant condition' on the SAE form.
This must be done irrespective or whether an adverse event has occurred and within 24 hours of awareness of pregnancy. The information submitted should include the anticipated date of delivery (see below for information related to induce termination of pregnancy).

The investigator will follow the subject until completion of the pregnancy or until pregnancy termination (i.e., induced abortion) and then notify the IELSG Operation Office of the outcome within 5 days. The investigator will provide this information as a follow up. The reason(s) for an induced abortion must be specified. If the outcome of the pregnancy meets the criteria for immediate classification as a serious adverse event (i.e., spontaneous abortion, stillbirth, neonatal death, or congenital anomaly [including that in an aborted fetus]), the investigator should follow up procedures for reporting serious adverse events and report the event to the IELSG Operation Office. In the case of a live birth, the "normality" of the newborn can be assessed at the time of birth (i.e., no minimum follow-up period of presumably normal infant must pass before an Exposure in Utero can be completed). The "normality" of an aborted fetus can be assessed by gross visual inspection unless pre-abortion laboratory finding are suggestive of a congenital abnormality.

Additional information about pregnancy outcomes that are classified as serious adverse events follows:

- "Spontaneous abortion" includes miscarriage and missed abortion.
- All neonatal death that occurs within 1month of birth should be reported, without regard to causality, as serious adverse events. In addition, any infant death after 1 month that the investigator assesses as possibly related to the in utero exposure to the investigational medication should also be reported.

9.2.2 Exposure During Lactation

An exposure during lactation occurs if an infant or child may have been exposed through breast milk to the study drug during breastfeeding by a female taking the study drug. Information regarding exposure during lactation is submitted to the IELSG Operation Office on a SAE Report Form within 24 hours of awareness of the exposure. Appropriate follow-up is required to determine the occurrence an outcome of any adverse event in the infant.

9.2.3 Serious Adverse Drug Reactions (SADRs)

All SAEs suspected to be related (see above 9.1.1) to the trial treatment are defined as Serious Adverse Drug Reactions (SADRs).

9.2.4 Suspected Unexpected Serious Adverse Reactions (SUSARs)

All SAE that are:
- suspected related to the study drug (therefore SADRs) AND
- not described in the protocol or in reference documents (e.g. Investigators' Brochure, Product Information/Summary of product characteristics) are defined as Suspected Unexpected Serious Adverse Reactions (SUSARs) and qualify for expedited reporting (see 9.3).

9.3 Procedures for AE, SAE and SUSAR Reporting

Patients will be instructed by the investigator to report the occurrence of any AE. The investigator assesses and records all AEs observed during the AE reporting period (i.e. from inclusion until 30 days after end of treatment). AEs are coded with the NCI Common Terminology Criteria for Adverse Events (CTCAE) v4.0, and assigned a grade (from 1 = mild to 5 = death related to AE) as well as a relationship (suspected vs. not suspected) to trial treatment. Any SAE must be reported by completing the "SAE form" (CRF) on line within 24 hours upon knowledge of the event (https://openclinica.eoc.ch/openClinica).

SAE reporting period is from trial registration until 30 days after end of experimental treatment.
The SAE outcome must be reported within 2 weeks after definitive assessment

The investigator, according to local regulations, will inform local authorities (ethics committee)
The physician responsible for patient care should organize any supplementary investigation of SAEs based on the clinical judgment on the likely causing factors. This means seeking a further opinion from a specialist in the field of the adverse event. If a patient dies, any post mortem finding including histopathology must be provided.
The sponsor (IELSG) shall be responsible for ensuring that any SAEs are appropriately reported to the relevant health authorities according to applicable laws and regulations in each country where the Study will be conducted and to perform any additional activities.

Reporting of SUSAR (suspected unexpected serious adverse reactions)
Events that fall into this category must be reported within 24 hours of occurrence using the SAE Report Form (see above) in the first instance. Then the reporter PI will be asked to fill in the appropriate CIOMS form (provided upon occurrence by IELSG Operation office).
It is the legal requirement of the sponsor to report SUSARs to the Competent Authorities and Ethics Committees (fatal or life-threatening within 7 days, nonfatal and non life-threatening within 15 days).

International (IESLG) Responsibilities

The Sponsor IELSG will also provide the sites involved in the clinical trial with a copy of the SUSARs reports and of the annual periodic safety report (DSUR) at the time of submission to the Regulatory Authorities and Ethics Committees

10 CLINICAL EVALUATION, LABORATORY TESTS AND FOLLOW-UP

10.1 Pretreatment

The following investigations have to be performed before registration (see the STUDY FLOW CHART to the detailed timelines of each assessment):

1. Physical & Neurological examination with ECOG performance status
2. Haemogram serum profile
3. Biochemical serum profile (including creatinine clearance and LDH)
4. Viral markers (HIV, HBV, HCV, CMV, HHV6-8, and parvovirus)*
5. Echocardiography with determination of LVEF in %
6. Respiratory volumes assessment with diffusion capacity measurements
7. Enhanced neck, thorax and abdomen (whole-body) CT scan
8. Ultrasonography of testes in men older than 60 years (or testicular involvement is suspected)
9. Bone marrow aspirate and/or biopsy
10. Gadolinum-enhanced whole-brain MRI
11. CSF cytology examination (cell count), physicochemical characterization (including protein concentration) and flow cytometry according to institutional guidelines
12. Ophthalmologic evaluation (including slit-lamp examination)
13. ^{18}FDG-PET

*with the most reliable method according to the Institutional Guidelines.

10.2 During treatment

A clinical and neurological evaluation should be performed immediately before the administration of the next chemotherapy course. Haemogram and biochemical blood profile will be performed before every chemotherapy course, and according to clinical requirements between the 7th and the 21st day of each course. CMV reactivation should be monitored during each cycle with the frequency and method adopted according to the Institutional Guidelines. Contrast-enhanced whole-body CT scan, gadolinium-enhanced MRI of the brain, CSF examination, FDG-PET, and ophthalmologic examination will be repeated every two chemoimmunotherapy courses if positive at baseline. Bone marrow biopsy will be performed after the conclusion of induction chemoimmunotherapy and after ASCT when positive at diagnosis, if clinically indicated.

10.3 After the end of the treatment

If the patient has not experienced progression, the disease should be assessed as follow: clinical examination, haemogram, biochemical blood profile, Gadolinium-enhanced brain MRI and contrasted total-body CT scan will be repeated every 3 months for 2 years, and then every 6 months for 3 years

10.4 After the progression of the disease

At relapse or progressive lymphoma, patients will be assessed with clinical examination, neurological evaluation, performance status, haemogram, serum chemistry, CSF cytology examination, slit lamp examination, ^{18}FDG-PET, brain MRI, bone marrow aspirate/biopsy, whole body CT scan. The patient should be followed every two months for survival or according to clinical requirements in the case a salvage therapy will be performed.

STUDY FLOW CHART

	Baseline[!]	During Treatment		After treatment	
	Within 14 days prior to treatment	Start of each cycle	Days 7- 21 of each cycle	Follow-up	At relapse
Medical history	X				
Clinical examination	X	X		X	X
Neurological evaluation	X	X		X	X
Performance status	X	X		X	X
Haemogram *	X[$]	X	***	X	X
Serum Chemistry^	X[$]	X	***	X	X
Creatinine clearance	X	X			
CSF cytology examination	X	X[**]			X
Slit lamp examination	X	X			X
^{18}FDG-PET	X	X[**]			X
Brain MRI	X	X[**]		X	X
Assessment target lesions	X[£]	X[**]			
Bone marrow aspirate/biopsy	X	X[****]			X
Whole body CT scan	X	X**		X	X
Viral markers (HIV, HBV, HCV, CMV, HHV6-8, and parvovirus)	X[§]	X[ç]			
Echocardiography with determination of LVEF in %	X				
Respiratory volumes assessment with diffusion capacity measurements	X				
Ultrasonography of testes	X[+]				
Pregnancy test (if applicable)	X^^				

! Baseline assessments performed within 14 days of treatment do not need repeating prior to cycle 1
* Hematology includes white blood cells, neutrophils, platelets and hemoglobin counts
^ Serum chemistry includes creatinine, bilirubin, alkaline phosphatase, LDH, AST, ALT, GGT
§ With the most reliable method according to the Institutional Guidelines.
+ In men older than 60 years (or testicular involvement is suspected)
$ To be performed within 7 days prior to start MATRIX
£ To be performed within 3 weeks prior to start MATRIX
ç Interval and methods of CMV reactivation monitoring during chemotherapy will follow Institutional Guidelines.
** These procedures will be performed every two chemotherapy courses to assess tumor response (see item 11.1.1 for details), and after the conclusion of induction chemoimmunotherapy and after ASCT.
# Slit-lamp exam and ophthalmoscopy should be performed every two courses only if positive at baseline.
*** These tests will be performed according to clinical requirements between day 7-21
**** If clinically indicated. Bone marrow biopsy will be performed after the conclusion of induction chemoimmunotherapy and after ASCT when positive at diagnosis. Once achieved CR, bone marrow biopsy is not more needed, with the exception of relapse, suspicion of marrow infiltration or other clinical requirements.

^^pregnancy test to be repeated within 7 days of treatment start date

11 EFFICACY ASSESSMENT

11.1 Criteria of evaluation

11.1.1 Schedule and methods of disease evaluation

Response of CNS and systemic lesions will be assessed after the 2nd, 4th and 6th courses of chemoimmunotherapy and 30-45 days after ASCT.
Response assessment every two courses will include enhanced brain MRI, CSF examination, contrasted thorax-abdomen CT scan, and PET. CSF examination will include the same exams performed at baseline. Slit-lamp exam and ophthalmoscopy should be performed every two courses only if positive at baseline; bone marrow biopsy will be performed after the conclusion of induction chemoimmunotherapy (i.e. before conditioning) and after ASCT when positive at trial registration, in the suspicion of relapsing or progressive lymphoma or in case of clinical requirement. Response assessment and definition will follow the revised response criteria for malignant lymphoma (17).
During follow-up, the disease will be evaluated by enhanced brain MRI and contrasted neck-thorax-abdomen(whole-body) CT-scan every three months for the first two years, every six months during the 3rd, 4th and 5th year of follow-up. Patients discontinuing therapy without any lymphoma progression should not receive any other cancer treatment, given this is not against patient's interest. CSF examination (as at baseline) will be performed only at failure (relapsing or progressive disease) or in the case of CSF dissemination suspicion.

11.1.2 Definition of response

Response assessment will be based on measurable change in tumour size:
Complete response: disappearance of all evidence of lymphoma
Partial response: ≥50 % decrease in tumour size.
Progressive disease: ≥25 % increase in tumour size or any new localization on any follow-up scan
Stable disease: all other situations.

The best response recorded from the start of the treatment until disease progression will be considered. Response of CNS and extra-CNS lesions will be recorded separately in the "ad hoc" CRF. Patients with bidimensional but not measurable disease who show tumour reduction during chemotherapy will be treated as patients with partial response.
In case of positive CSF at diagnosis, a reduction >50% of cells number will be considered as partial response, while a reduction <50% will be considered as stable disease.

11.1.3 Duration of the response

The duration of response will be measured from the date of maximum response to the date of objective progression, death for any cause or last visit of follow-up.

11.1.4 Patient without any response assessment

All eligible patients will be considered for response evaluation.
Patients who die before response assessment will be considered as 'early deaths', and classified according to the cause of death: progression, toxicity or disease and drug unrelated.
Patients who discontinue treatment before response assessment will be considered as 'early discontinuation' due to progression, toxicity or disease and drug unrelated.

Patients stopping treatment with an unconfirmed response or a too short stabilization will be considered as ‘early discontinuation’, unless the response or stabilization is later confirmed without any further treatment.
Patients not evaluated for any reason (lost to follow-up, refusal…) will be considered as ‘not assessed’.

11.2 Transplantation Efficacy measures

11.2.1 Characterization of the stem cell graft

Samples of the stem cell graft shall be characterized with respect to the number of CD34 positive cells per kg body weight of the recipient. The number of transplanted CD34 positive cells per kg body weight of the recipient shall be recorded in the CRF. If the transplant was cryopreserved, the number of viable CD34 positive cells has to be determined after thawing and documenting. The goal is to transplant 5 to 10 x 106 CD34+ cells/kg BW recipient from peripheral blood stem cells.

11.2.2 Definition of engraftment

Leukocyte engraftment: first of 3 consecutive days with leukocyte count >1 x 10^9 /L in the peripheral blood.
Neutrophil engraftment: first of 3 consecutive days with ANC >0.5 x 10^9/L in the peripheral blood.
Platelet engraftment: first of 3 consecutive days with platelets >20 x 10^9 /L and >50 x 10^9 /L without platelet transfusion.

11.2.3 Evaluation of engraftment

The engraftment will be evaluated on the hand of daily counts for leukocytes, neutrophils and platelets from day -6 until all the following requirements are met, but at least until day +21, and on day +24 and +28. In case of delayed regeneration of thrombopoiesis (>28 days after transplantation), weekly assessments are sufficient.

11.2.4 Documentation of transfusions (platelet and erythrocyte concentrates)

The number of platelet and erythrocyte concentrates required from day -6 until day 100 must be recorded.

11.2.5 Screening

Patients indicated for ASCT will be checked for their general eligibility for the transplant procedure.
These examinations are not part of the study protocol.

11.2.6 Initial examinations

All initial examinations performed according to clinical standards are required for patients enrolled into high dose-density schedule treatment.

Within 2 weeks before day 0 (date of transplant)

- Inclusion criteria
- HIV, HBV and HCV tests
- Pregnancy test for women of childbearing potential (within 2 weeks before day –6
- Creatinine clearance
- Blood chemistry (within 2 weeks before day –6)
- Total bilirubin

- Urine analysis (within 2 weeks before day –6)
- Medical history
- Physical examination including ECOG Performance Status
- Blood count (within 2 weeks before day –6)
- Automated neutrophil count (within 2 weeks before day –6)
- Differential blood count (within 2 weeks before day –6)
- Disease status in bone marrow and peripheral blood

Physical examination shall be performed according to the hospital standard and should include all organ systems (including a basic neurological examination), blood pressure, pulse, weight, body temperature (axillary), performance status, and interview for adverse events.

11.2.7 Interim examinations

Day 0 to Day +30

- Physical examination
- Weight (daily until day +21, day +24)
- Adverse events and Serious adverse events evaluation, continuous documentation
- Serum bio-chemistry at day 0; +2; +7; +10; +14; +21; +30
- Determination of blood count / automated neutrophil count daily from day 0 until all the following requirements are met, but at least until day +21, and on day +30 (for definitions refer to chapter 8.1.2): leukocyte, neutrophil and platelet engraftment (in case of delayed regeneration of thrombopoiesis (> 28 days) weekly assessments are sufficient)
- Recording of concomitant medications
- Documentation of platelet / erythrocyte transfusions

11.2.8 Appropriateness of measures

All measures are standard examinations, which are routinely performed in transplantation.

11.2.9 Primary Engraftment efficacy

The most direct effect of a conditioning regimen prior to autologous transplantation is to allow fast and reliable engraftment of the transplanted haematopoietic stem cells. Therefore, the conditional probability of neutrophil engraftment at day +14 was chosen as primary efficacy variable.

11.3 Definition of Endpoints

11.3.1 Primary endpoint

The primary end point of the study is 1-year progression-free survival (PFS).
PFS for all patients is defined as the interval between the time of entry onto trial and failure (relapsing or progressive disease), death from any cause or date of the last visit of follow-up.

11.3.2 Secondary endpoints

The secondary endpoints of the study are:

- Complete remission rate before autologous stem cell transplantation
- Response duration: for all patients it is defined as the time from the first assessment that documents the response to the date of relapse, date of death from any cause or date of the last visit of follow-up
- Overall survival (OS): for all patients is defined as the time from entry onto trial until death from any cause or date of the last visit of follow-up.
- Safety for all the patients is defined as the assessment of the AEs according to NCI CTCAE V4.0

12 STATISTICAL METHODS

12.1 Statistical design and sample size

The Fleming design will be used.
The maximum 1-year PFS rate considered of low interest is 50% (P0) [Korfel et al. Haematologica 2013 (18)] and the minimum 1-year PFS rate considered of interest is 65% (P1). In order to detect such a difference a total number of 69 patients is required (one-sided test, type I error 5% and power 80%), with a drop-out of 10%, 76 patients will be needed. If at least 41 patients will be progression-free survivors at 1 year, the strategy will be considered effective.
All primary analyses are based on intention to treat, where all registered patients are included with the exception of patients who post-hoc objectively do not meet the eligibility criteria at the time of registration.
Differences in response rate among subgroups of interest will be tested using the test chi-square. Survival curves will be estimated using the Kaplan-Meier method and the Log rank test will be used to compare outcome of the different subgroups of interest. Independent association between studied variables and survival will be tested using the Cox proportional hazard model.

12.2 Preliminary safety analysis

A preliminary safety analysis will be performed after recruitment of the first 5 patients to address feasibility and introduce timely changes in treatment schedule. This analysis will be performed by trial chairpersons, who will establish the early stopping rule to be used and the frequency and timing of further safety analyses

13 PATHOLOGY

13.1 Local pathology

The local pathologist of each center participating in this trial has to be informed by the local investigator about the trial protocol, particularly about data and sample processing.

13.2 Pathology review

After examination, available pathological material must be “anonymised” and sent for central pathology review to the IELSG Coordinating Center (IOSI, Ospedale San Giovanni, CH 6500 Bellinzona, Switzerland - ielsg@eoc.ch- phone +41 91 811 9040), together with a copy of the original pathology report(s) including diagnosis, classification and immunophenotyping. An IELSG panel of expert pathologists will review all cases under the supervision of Dr. Maurilio Ponzoni (Unit of Lymphoid Malignancies, Pathology Unit, San Raffaele Scientific Institute, Milan, Italy).

14 ETHICAL CONSIDERATIONS

The IELSG will act as sponsor of this international multi-centre trial which will be conducted in accordance with the Declaration of Helsinki, the Guidelines of Good Clinical Practice issued by the International Conference on Harmonisation (ICH) and the appropriate regulatory requirement(s).
The IRB/EC of each participating center will review all appropriate study documentation in order to safeguard the rights, safety and well-being of the patients. The study will only be conducted at sites with IRB/EC approval and competent authority acceptance.
Therefore before planning to enter any patients into this trial, the investigator has to make sure that his center has been authorized/activated. Copies of the approval letters will keep on file at the IELSG.
The investigator is responsible for ensuring that the clinical study is conducted in accordance with the protocol, current ICH guidelines on Good Clinical Practice (GCP), and applicable regulatory requirements.
The patient's consent to participate in the study must be obtained for all cases, after a written full explanation has been given of the treatment and before performance of any study-related activity. Patient Information Form and Informed Consent Forms must be approved by IELSG and must be finalized by the principal investigator in each country/institution following the requirements of the local Regulatory Authorities and ECs.
The right of a patient to refuse to participate without giving reasons must be respected.
After the patient has entered the trial, the clinician must remain free to give alternative treatment to that specified in the protocol at any stage if it is felt to be in the patient's best interests. The reason for giving such alternative treatment should be recorded and the patient should remain in the study for the purposes of follow-up and data analysis.
Similarly, the patient must remain free to withdraw consent at any time without giving reasons and without prejudicing further treatment.

15 ADMINISTRATIVE CONSIDERATIONS

15.1 Monitoring and auditing

The investigator/institution should make available for direct access all requested trial-related records and should permit monitoring and auditing by the sponsor, and inspection by ethics committees and the appropriate regulatory authority(ies).

15.2 Data protection and archiving

Patient confidentiality will be maintained according to applicable legislation. Patients must be informed of, and agree to, data transfer and handling, in accordance with data protection law.
Patient data will be pseudonymised: each patient in the study will be uniquely identified by a code which is a combination of 2-digit trial number, followed by 3-digit centre number and 3-digit subject number.
The trial and the center numbers are assigned by IELSG Operation Office. Upon signing the informed consent form, a patient number is assigned to every single patient by the PI, using consecutive numbers (e.g. 001, 002, 003,...).
The decryption key ("*subject identification log")* must be kept in a safe place by the investigator, at the trial centre, and only made accessible to authorized and authenticated persons.

All information collected during the trial project must be stored correctly and for the appropriate length of time (at least 10 years).

15.3 Quality control and quality assurance

Several procedures guarantee quality of trial conduct:

- Reviews of protocol and forms according to standard operating procedures
- Requirements for principal investigators for participation: signed and dated CV and trial-specific agreement
- Validation of database and statistical analysis
- CRFs will be checked; queries will be issued in case of inconsistencies.
- Data review by the trial chair or a delegated person (all CRFs will be reviewed and checked on medical content)
- Safety monitoring
- An authorization list must be kept at the center
- Accountability of trial drugs
- The trial will be monitored
- Internal audit of the trial
- Other (disclosures of conflicts of interest and financial support)

16 PUBLICATION

The results of this study will be submitted for publication in peer reviewed journals and for presentation at appropriate scientific meetings.

IELSG wants to recognize the contributions of all individuals who take part in the preparation of the study, the analysis of data, including patients.

The study chair on the basis of the statistical analysis will write the final publication of the trial results.

The study chair should make the final decision regarding authorship and order of authors on a manuscript; this authors' list should be approved by the Director of the IELSG Operation Office and by the President of the IELSG Board of Directors.

In principle, the study chair will be the first/last author of any publication and other contributors will be included as authors according to their input into the study:

- the investigators who have included more than 5% of the eligible patients in the trial by order of the number of registered patients per center
- the study statistician
- additional members who contribute significantly to study design, analysis, management of data, trial coordination, manuscript writing
- representatives of other disciplines (e.g., pathologists, molecular biologists and others)
- ideally, each otherwise unrepresented country or co-operative group, which have enrolled patients, should have the opportunity to include one author (this may be dealt with highly accruing sites)
- additional authors can be added (e.g., representatives of centers contributing significantly to the patient population of the study).

Anyhow, all the contributors who do not meet the criteria for authorship should be listed in an appendix to the manuscript or in the acknowledgments.

The acknowledgments' list will include the names of all participating institutions together with the corresponding names of the principal investigators.

No publication can occur without agreement of the study chair, IELSG Operation Office, and Board of Directors. Case reports on therapeutic features involving patients registered in a IELSG trial are strongly discouraged. However, participating investigators may report their own experience on patients registered in this trial at scientific meetings or journals with permission of the IELSG and of the study chair without disclosing the overall results of the study. If this is foreseen, the investigator agrees to submit all their abstracts or manuscripts to the study chair and to the IELSG Operation Office prior to submission. This allows the sponsor and the study chair to ascertain that the communication will not undermine the value of the whole study and to provide comments based on information that may not be yet available to all the investigators; a written reply will then be sent by the study coordinator to the investigators, within 2 weeks.

17 REFERENCES

(1) Villa D, Connors JM, Shenkier TN, Gascoyne RD, Sehn LH, Savage KJ. Incidence and risk factors for central nervous system relapse in patients with diffuse large B-cell lymphoma: the impact of the addition of rituximab to CHOP chemotherapy. Ann Oncol 2010;21(5):1046-1052.

(2) Boehme V, Zeynalova S, Kloess M, Loeffler M, Kaiser U, Pfreundschuh M, et al. Incidence and risk factors of central nervous system recurrence in aggressive lymphoma--a survey of 1693 patients treated in protocols of the German High-Grade Non-Hodgkin's Lymphoma Study Group (DSHNHL). Ann Oncol 2007;18(1):149-157.

(3) Mappa S, Marturano E, Licata G, Frezzato M, Frungillo N, Ilariucci F, et al. Salvage chemoimmunotherapy with rituximab, ifosfamide and etoposide (R-IE regimen) in patients with primary CNS lymphoma relapsed or refractory to high-dose methotrexate-based chemotherapy. Hematol Oncol 2012; DOI: 10.1002/hon.2037.

(4) Kasenda B, Schorb E, Fritsch K, Finke J, Illerhaus G. Prognosis after high-dose chemotherapy followed by autologous stem-cell transplantation as first-line treatment in primary CNS lymphoma--a long-term follow-up study. Ann Oncol 2012;23(10):2670-2675.

(5) Ferreri AJ, Reni M, Foppoli M, Martelli M, Pangalis GA, Frezzato M, et al. High-dose cytarabine plus high-dose methotrexate versus high-dose methotrexate alone in patients with primary CNS lymphoma: a randomised phase 2 trial. Lancet 2009;374: 1512–1520.

(6) Ferreri AJ, Reni M, Pasini F, Calderoni A, Tirelli U, Pivnik A, et al. A multicenter study of treatment of primary CNS lymphoma. Neurology 2002;58(10):1513-1520.

(7) Reni M, Ferreri AJ, Guha-Thakurta N, Blay JY, Dell'Oro S, Biron P, et al. Clinical relevance of consolidation radiotherapy and other main therapeutic issues in primary central nervous system lymphomas treated with upfront high-dose methotrexate. Int J Radiat Oncol Biol Phys 2001;51(2):419-425.

(8) Coiffier B, Lepage E, Briere J, Herbrecht R, Tilly H, Bouabdallah R, et al. CHOP chemotherapy plus rituximab compared with CHOP alone in elderly patients with diffuse large-B-cell lymphoma. N Engl J Med 2002;346(4):235-242.

(9) Batchelor TT, Grossman SA, Mikkelsen T, Ye X, Desideri S, Lesser GJ. Rituximab monotherapy for patients with recurrent primary CNS lymphoma. Neurology 2011;76(10):929-930.
(10) Shah GD, Yahalom J, Correa DD, Lai RK, Raizer JJ, Schiff D, et al. Combined immunochemotherapy with reduced whole-brain radiotherapy for newly diagnosed primary CNS lymphoma. J Clin Oncol 2007;25(30):4730-4735.

(11) Kaijser GP, De Kraker J, Bult A, Underberg WJ, Beijnen JH. Pharmacokinetics of ifosfamide and some metabolites in children. Anticancer Res 1998;18(3B):1941-1949.

(12) Relling MV, Mahmoud HH, Pui CH, Sandlund JT, Rivera GK, Ribeiro RC, et al. Etoposide achieves potentially cytotoxic concentrations in CSF of children with acute lymphoblastic leukemia. J Clin Oncol 1996;14(2):399-404.

(13) Arellano-Rodrigo E, Lopez-Guillermo A, Bessell EM, Nomdedeu B, Montserrat E, Graus F. Salvage treatment with etoposide (VP-16), ifosfamide and cytarabine (Ara-C) for patients with recurrent primary central nervous system lymphoma. Eur J Haematol 2003;70(4):219-224.

(14) Takasu S, Wakabayashi T, Kajita Y, Hatano N, Hatano H, Usui T, et al. Effectiveness of DeVIC chemotherapy for recurrent primary central nervous system lymphoma. No Shinkei Geka 2000;28(9):789-794.

(15) Motomura K, Natsume A, Fujii M, Ito M, Momota H, Wakabayashi T. Long-term survival in patients with newly diagnosed primary central nervous system lymphoma treated with dexamethasone, etoposide, ifosfamide and carboplatin chemotherapy and whole-brain radiation therapy. Leuk Lymphoma 2011;52(11):2069-2075.

(16) Ferreri AJ, Dell'Oro S, Foppoli M, Bernardi M, Brandes AA, Tosoni A, et al. MATILDE regimen followed by radiotherapy is an active strategy against primary CNS lymphomas. Neurology 2006;66(9):1435-1438.

(17) Cheson BD, Pfistner B, Juweid ME, Gascoyne RD, Specht L, Horning SJ, et al. Revised response criteria for malignant lymphoma. J Clin Oncol. 2007;25(5):579-586.

(18) Korfel A, Elter T, Thiel E, Hänel M, Möhle R, Schroers R, et al. Phase II study of central nervous system (CNS)-directed chemotherapy including high-dose chemotherapy with autologous stem cell transplantation for CNS relapse of aggressive lymphomas. Haematologica 2013;98(3):364-370.
